# GOT1 inhibition promotes pancreatic cancer cell death by ferroptosis

**DOI:** 10.1038/s41467-021-24859-2

**Published:** 2021-08-11

**Authors:** Daniel M. Kremer, Barbara S. Nelson, Lin Lin, Emily L. Yarosz, Christopher J. Halbrook, Samuel A. Kerk, Peter Sajjakulnukit, Amy Myers, Galloway Thurston, Sean W. Hou, Eileen S. Carpenter, Anthony C. Andren, Zeribe C. Nwosu, Nicholas Cusmano, Stephanie Wisner, Nneka E. Mbah, Mengrou Shan, Nupur K. Das, Brian Magnuson, Andrew C. Little, Milan R. Savani, Johanna Ramos, Tina Gao, Stephen A. Sastra, Carmine F. Palermo, Michael A. Badgley, Li Zhang, John M. Asara, Samuel K. McBrayer, Marina Pasca di Magliano, Howard C. Crawford, Yatrik M. Shah, Kenneth P. Olive, Costas A. Lyssiotis

**Affiliations:** 1 Department of Molecular & Integrative Physiology, University of Michigan, Ann Arbor, MI USA; 2 Graduate Program in Chemical Biology, University of Michigan, Ann Arbor, MI USA; 3 Graduate Program in Cancer Biology, University of Michigan, Ann Arbor, MI USA; 4 Immunology Graduate Program, University of Michigan, Ann Arbor, MI USA; 5 Department of Internal Medicine, Division of Gastroenterology and Hepatology, University of Michigan, Ann Arbor, MI USA; 6 Department of Biostatistics, School of Public Health, University of Michigan, Ann Arbor, MI USA; 7 Rogel Cancer Center, University of Michigan, Ann Arbor, MI USA; 8 Medical Scientist Training Program, University of Texas Southwestern Medical Center, Dallas, TX USA; 9 Division of Digestive and Liver Diseases, Department of Medicine, Columbia University Medical Center, New York, NY USA; 10 Herbert Irving Comprehensive Cancer Center, Columbia University Medical Center, New York, NY USA; 11 Department of Pathology, Columbia University Medical Center, New York, NY USA; 12 Division of Signal Transduction and Mass Spectrometry Core, Beth Israel Deaconess Medical Center, Boston, MA USA; 13 Department of Medicine, Harvard Medical School, Boston, MA USA; 14 Children’s Medical Center Research Institute and Department of Pediatrics, University of Texas Southwestern Medical Center, Dallas, TX USA; 15 Department of Surgery, University of Michigan, Ann Arbor, MI USA; 16 Department of Cell and Developmental Biology, University of Michigan, Ann Arbor, MI USA

**Keywords:** Cancer metabolism, Pancreatic cancer

## Abstract

Cancer metabolism is rewired to support cell survival in response to intrinsic and environmental stressors. Identification of strategies to target these adaptions is an area of active research. We previously described a cytosolic aspartate aminotransaminase (GOT1)-driven pathway in pancreatic cancer used to maintain redox balance. Here, we sought to identify metabolic dependencies following GOT1 inhibition to exploit this feature of pancreatic cancer and to provide additional insight into regulation of redox metabolism. Using pharmacological methods, we identify cysteine, glutathione, and lipid antioxidant function as metabolic vulnerabilities following GOT1 withdrawal. We demonstrate that targeting any of these pathways triggers ferroptosis, an oxidative, iron-dependent form of cell death, in GOT1 knockdown cells. Mechanistically, we reveal that GOT1 inhibition represses mitochondrial metabolism and promotes a catabolic state. Consequently, we find that this enhances labile iron availability through autophagy, which potentiates the activity of ferroptotic stimuli. Overall, our study identifies a biochemical connection between GOT1, iron regulation, and ferroptosis.

## Abstract (web summary)

The aspartate aminotransaminase GOT1 is important for maintaining redox balance. Here, the authors show that inhibition of GOT1 in pancreatic cancer cells leads to cell death via ferroptosis.

## Introduction

**Fig. 1:**
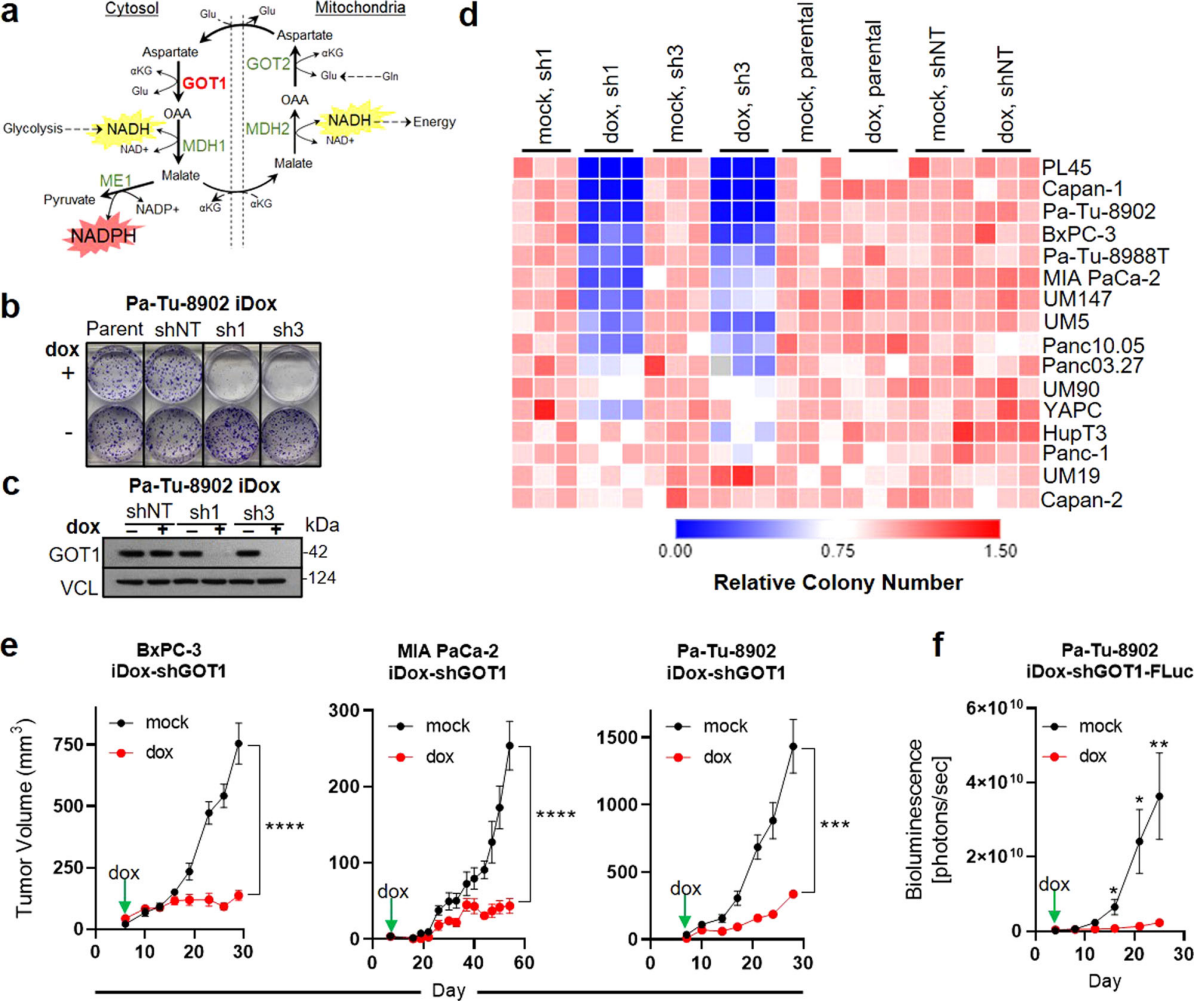
PDA requires GOT1 for cellular proliferation and tumor growth. **a** Malate-aspartate shuttle model. **b**, **c** Colony formation and immunoblot analysis of Pa-Tu-8902 cells stably expressing iDox-shRNA constructs following 10 days GOT1 knockdown. shRNAs target the coding region of GOT1 (sh1), or the 3′UTR region of GOT1 (sh3). Parental (parent) and scramble (shNT) conditions are also displayed. Vinculin (VCL) was used as a loading control. Immunoblot shown in (**c**) is representative of three independent experiments. **d** Relative colony number across a panel of PDA cell lines, *n* = 3 biological replicates. **e** Subcutaneous xenograft tumors from three PDA cell lines. Treatment with dox (red) or vehicle (black) (BxPC-3 mock-treated *n* = 8 mice; dox *n* = 6 mice; ****P = < 0.0001, MIA PaCa-2 *n* = 6 mice each group; ****P < 0.0001, Pa-Tu-8902 *n* = 6 mice each group; ***P = 0.0008). **f** Orthotopic xenograft tumor growth from Pa-Tu-8902 iDox-shGOT1 stable cells expressing firefly luciferase (FLuc) *n* = 5 and *n* = 6 mice were used for vehicle and dox cohorts, respectively; *P = 0.0138, *P = 0.0165, **P = 0.0102. Data shown represent biological replicates examined over one experiment and reproduced in two independent experiments. Error bars represent mean ± SD. Source data are provided as a Source Data file. Gln glutamine, αKG alpha-ketoglutarate, Glu glutamate, OAA oxaloacetate, NADH nicotinamide adenine dinucleotide, NADPH nicotinamide adenine dinucleotide phosphate, GOT glutamic oxaloacetic transaminase, MDH malate dehydrogenase, ME malic enzyme.

Pancreatic ductal adenocarcinoma (PDA) cells exhibit extensive metabolic reprogramming to support survival and growth^1,2^. Our previous work demonstrated that PDA rewire the malate-aspartate shuttle to generate reduced nicotinamide adenine dinucleotide phosphate (NADPH), a major currency for biosynthesis and redox balance (Fig. 1a)^3^. The activity of this non-canonical pathway was orchestrated by mutant KRAS through control of the expression of the cytosolic aspartate aminotransaminase (GOT1). Because GOT1 is dispensable in non-malignant cells, but is essential for redox balance and proliferation in PDA^3^, GOT1 and its adjacent metabolic network could represent attractive therapeutic targets.

The canonical functions of the malate-aspartate shuttle and GOT1 are to transfer reducing equivalents in the form of NADH from the cytosol into the mitochondria to facilitate oxidative phosphorylation (OxPHOS). In PDA, we found the mitochondrial aspartate aminotransaminase (GOT2) is the primary anaplerotic source for alpha-ketoglutarate (αKG) and generates aspartate. Aspartate is then transferred to the cytosol and transaminated to produce oxaloacetate (OAA) by GOT1. OAA is reduced to malate by cytosolic Malate Dehydrogenase 1 (MDH1) and is then oxidized by Malic Enzyme 1 (ME1) to generate NADPH, which is utilized to support redox balance and proliferation in PDA^3^.

Thus, in an effort to target this rewired metabolic pathway and to further understand its biological role, in this study, we focused on GOT1. Here, we analyzed GOT1 inhibition across a large panel of PDA cell lines and specimens. We show that sensitivity to GOT1 knockdown varies among the cultures in this panel, is dispensable in non-transformed human lines, and that GOT1 inhibition stunted growth in tumor models. In GOT1 responsive contexts, GOT1 inhibition blocked progression through the cell cycle, leading to cytostasis. Thus, we then sought to characterize metabolic dependencies following GOT1 withdrawal that could be exploited to selectively kill PDA. Examination of a targeted metabolic inhibitor library in GOT1 knockdown cells led to the discovery that exogenous cystine, glutathione, and lipid antioxidant machinery were essential for viability following chronic GOT1 suppression. GOT1 knockdown in combination with inhibitors of cystine import or lipid antioxidant machinery led to ferroptosis: an oxidative, non-apoptotic, and iron-dependent form of cell death^4,5^. We then determined that GOT1 withdrawal impaired mitochondrial OxPHOS, thereby promoting a catabolic state. Consequently, the cells increased labile iron pools which increased their susceptibility to ferroptosis.

## Results

### PDAs utilize GOT1 for cellular proliferation and tumor growth

To determine GOT1 utilization in PDA with temporal control, we employed our previously described doxycycline (dox)-inducible short-hairpin (sh)RNA reagents (iDox-sh) that target the coding and 3′UTR regions of GOT1 (sh1 and sh3), or scramble (shNT)^3,6^. shRNA activity was examined phenotypically by assessing colony formation and protein levels following dox treatment (Fig. 1b, c and Supplementary Fig. 1a, b). We also measured aspartate levels as a biochemical readout for GOT1 inhibition (Supplementary Fig. 1c). We then used these iDox-shRNA constructs to examine GOT1 sensitivity across a large panel of PDA lines and primary specimens (indicated with the UM# designation)^7^ (Fig. 1d). GOT1 knockdown significantly impaired colony formation in 12 of 18 cell lines in the panel (Fig. 1d). The response to GOT1 knockdown did not depend on the expression status of malate-aspartate shuttle enzymes (Supplementary Fig. 1d). Importantly, CRISPR/Cas9 mediated knockout of GOT1 blunted colony formation and proliferation (Supplementary Fig. 1e–g). To test the specificity of GOT1 against PDA, we extended our cell panel to non-transformed human lines. We found human pancreatic stellate cells (hPSC), human lung fibroblasts (IMR-90), and human non-transformed pancreatic exocrine cells (hPNE) were minimally affected upon GOT1 knockdown, in agreement with previous results, suggesting that this pathway may be dispensable in non-transformed cells (Supplementary Fig. 1h–i)^3,8^, highlighting a potential therapeutic window.

We then examined the effect of GOT1 inhibition on established PDA tumors. PDA cells were implanted subcutaneously into the flanks or orthotopically into the pancreas of immunocompromised mice and allowed to establish for 7 days prior to GOT1 inhibition. GOT1 sensitive cell lines exhibited profound growth inhibition upon induction of GOT1 knockdown with dox (Fig. 1e, f), consistent with previous studies^3,6^. Parallel studies with shNT tumors indicated that the effect was independent of dox (Supplementary Fig. 2a). GOT1 knockdown was demonstrated by immunoblot analysis on homogenized tumor tissue (Supplementary Fig. 2b) and biochemically via the induction of aspartate (Supplementary Fig. 2c). Tumor growth suppression was confirmed at the molecular level by a decrease in Ki-67, a marker for proliferation (Supplementary Fig. 2d). GOT1 knockdown tumors exhibited minimal staining for cleaved caspase 3, a marker for apoptosis. Thus, GOT1 inhibits tumor growth by inhibiting proliferation, rather than inducing cell death (Supplementary Fig. 2d).

To test the hypothesis that GOT1 inhibition is cytostatic, we examined the effect of GOT1 knockdown on cell cycle progression. Knockdown led to a higher distribution of cells in G1 phase versus the S and G2 phases, indicating that most cells are in G1 cell cycle arrest following 5 days of dox treatment (Supplementary Fig. 2e). The effect of GOT1 knockdown was reversible, as cells regained proliferative capacity upon removal of genetic inhibition (Supplementary Fig. 2f). Overall, PDA display a spectrum of sensitivity to GOT1, where GOT1 inhibition impairs proliferation without inducing cytotoxicity.

### Limiting exogenous cystine potentiates GOT1 inhibition

**Fig. 2:**
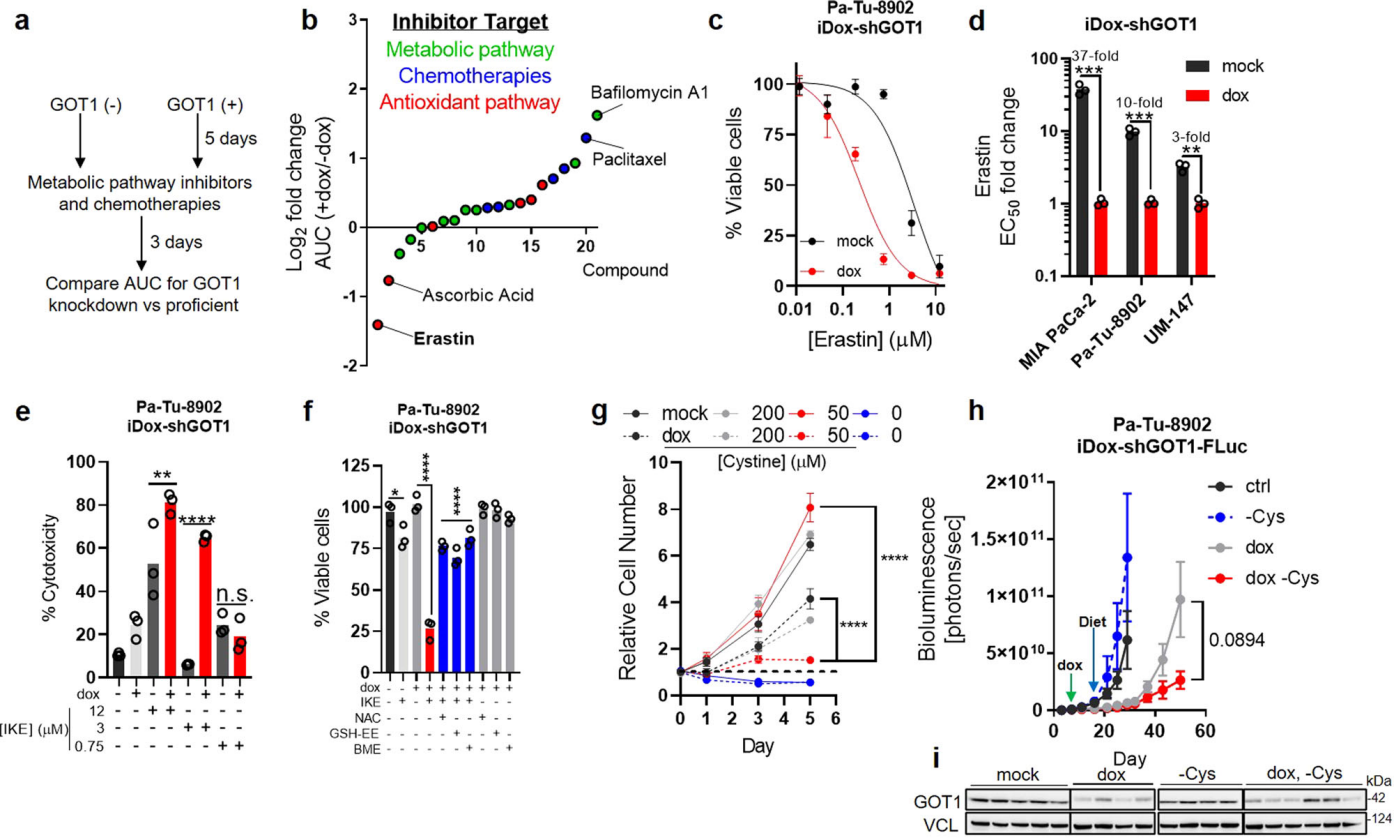
PDA requires cystine for viability and growth following GOT1 inhibition. **a** Screening strategy to identify metabolic dependencies following GOT1 suppression. **b** Log_2_ fold change in area under the curve (AUC) from cell viability dose response curves corresponding to each point, *n* = 3 biological replicates. **c** Cell viability dose response to erastin comparing mock (black) and GOT1 knockdown (red). **d** GOT1 sensitization represented as the fold change in the erastin EC_50_, *n* = 3 biological replicates, ***P = 0.000526, ***P = 0.000206, **P = 0.001204. **e** % Cytotoxicity following GOT1 knockdown and IKE (Imidazole ketone erastin) treatment for 24 h. Cytotoxicity was measured by LDH (Lactate dehydrogenase) release and normalized to a cell lysis control, *n* = 3 biological replicates, **P = 0.0044, ****P < 0.0001, n.s. *P* = 0.9838. **f** Cell viability of Pa-Tu-8902 iDox-shGOT1 after 5 days of GOT1 knockdown then 24 h of 750 nM IKE combined with the indicated conditions. 250 μM of N-acetyl-cysteine (NAC), 250 μM GSH-ethyl ester (GSH-EE), and 50 μM of beta-mercaptoethanol (BME) were used (*n* = 3 biological replicates), *P = 0.0254, ****P < 0.0001, ****P < 0.0001. **g** Proliferation following GOT1 knockdown and the indicated media conditions (*n* = 3 biological replicates), ****P < 0.0001, ****P < 0.0001. **h**, **i** Orthotopic xenograft tumor growth from Pa-Tu-8902 iDox-shGOT1 stable cell lines co-expressing firefly luciferase (FLuc) treated with vehicle (black, *n* = 6 mice), dox-containing food (gray, *n* = 6 mice), cysteine-free diet (-Cys) (blue, *n* = 5 mice), or dox-containing, cysteine-free food (red, *n* = 6 mice), n.s. *P* = 0.0894. GOT1 immunoblot (i) taken from endpoint tumors. Immunoblot in (**i**) is representative of two independent experiments. Error bars represent mean ± SD in **b**–**g** or mean ± SEM in (**h**). Two-tailed unpaired *T*-test or one-way ANOVA. Source data are provided as a Source Data file.

Because GOT1 inhibition is uniquely cytostatic in PDA relative to non-transformed cells, we sought to identify metabolic vulnerabilities induced by GOT1 knockdown that could be targeted to kill PDA. To test this, we examined the sensitivity of GOT1 knockdown PDA cells in response to panel of metabolism-targeted small molecules. Cells were subjected to 5 days of dox treatment to ensure GOT1 knockdown followed by 3 days of drug treatment (Fig. 2a).

GOT1 inhibition was protective when combined with some inhibitors, demonstrated by increased area under the curve values, a measure of drug sensitivity (Fig. 2b and Supplementary Fig. 3a). Three of the five top desensitizing agents were chemotherapies, in agreement with previous observations^6^. By contrast, GOT1 knockdown potentiated erastin sensitivity (Fig. 2b, c). Erastin is an inhibitor of the system x_c_^—^ cystine/glutamate antiporter, which transports cystine into cells in exchange for glutamate^4^. Cystine, the oxidized dimer of cysteine, is reduced to cysteine upon entering the cell where it can contribute to the synthesis of GSH and proteins, among numerous other biochemical fates. GOT1 knockdown potentiated erastin sensitivity in additional PDA cell lines (Fig. 2d and Supplementary Fig. 3b) and was independent of dox interference (Supplementary Fig. 3c). This GOT1 potentiating effect was phenocopied by the erastin analog imidazole ketone erastin (IKE) (Supplementary Fig. 3d), and IKE combination treatment led to partial cytotoxicity (Fig. 2e and Supplementary Fig. 3e, f). Treating GOT1 knockdown cells with nano-molar doses of erastin or IKE reduced cell numbers compared with single treatment arms (Supplementary Fig. 3g). Further, this could be reversed via supplementation with exogenous cysteine or GSH sources—i.e., N-acetyl cysteine (NAC), β-mercapto ethanol (BME), or cell permeable GSH-ethyl-ester (GSH-EE) (Fig. 2f and Supplementary Fig. 3h), consistent with the model that cystine import through system x_c_^—^ is essential to maintain GSH levels.

Previous studies have found cystine levels were limiting in PDA tumors^9,10^. Culturing cells in tumor-relevant cystine potentiated GOT1 knockdown in a time- and dose-dependent manner (Fig. 2g and Supplementary Fig. 4a, b), in agreement with our pharmacological studies. These results indicate PDA require exogenous cystine for growth and cell viability following GOT1 inhibition to aid cells in coping with redox stress^3,6^. In line with this observation, GOT1 inhibition led to increased intracellular cysteine and unaltered intracellular glutamate levels (Supplementary Fig. 4c). xCT protein levels were unchanged (Supplementary Fig. 4d), suggesting higher cystine uptake following GOT1 knockdown.

To target cysteine and GOT1 in vivo, we engrafted Pa-Tu-8902 iDox-shGOT1 cells engineered to express firefly luciferase (FLuc) into the pancreas, as in Fig. 1f. Tumors were allowed to establish for 7 days, and treatment arms were initiated by providing dox-containing food formulated with or without the non-essential amino acid cysteine. Tumors in the animals fed a cysteine-free diet grew at comparable rates to tumors in animals fed a control diet, and dox-treated tumors grew substantially slower than single-arm controls (Fig. 2h). Tumor growth and burden trended lower in the combination diet compared to dox-treated tumors (Fig. 2h, i and Supplementary Fig. 4e). Ki-67 staining revealed an effect on tumor proliferation in the dox-treatment groups (Supplementary Fig. 4f). Mice fed with a cysteine-free diet had lower cysteine in tumors compared to the control diet (Supplementary Fig. 4g), though cysteine levels were not significantly altered in tumors from the double treatment arms (Supplementary Fig. 4g). This suggests PDA tumors may acquire cysteine or antioxidants from the surrounding microenvironment when challenged by chronic cysteine deprivation. Overall, these data indicate that PDA cultures are sensitized to exogenous cystine withdrawal following GOT1 inhibition.

### Inhibiting GSH biosynthesis potentiates the growth inhibitory effects of GOT1 knockdown

**Fig. 3:**
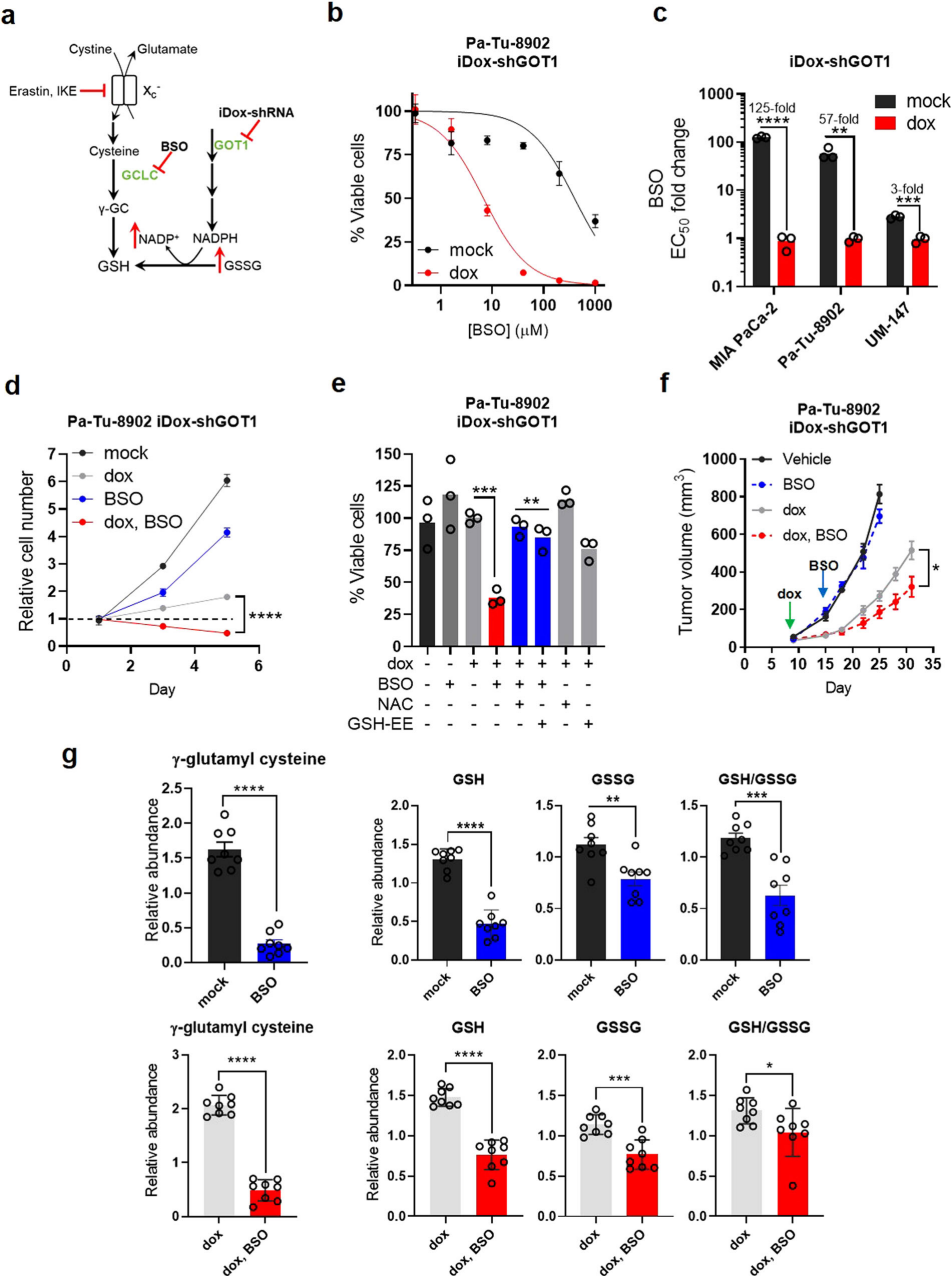
PDA require GSH synthesis for growth upon GOT1 suppression. **a** Scheme depicting GSH synthesis and metabolic changes following GOT1 inhibition. **b** Cell viability dose response, *n* = 3 biological replicates. **c** EC_50_ fold change across multiple PDA cell lines (****P = 0.000008, **P = 0.003905, ***P = 0.000359) following 5 days of GOT1 knockdown and BSO treatment, *n* = 3 biological replicates. **d** Proliferation (****P = < 0.0001) following 5 days of GOT1 knockdown and BSO treatment, *n* = 3 biological replicates. **e** Cell viability following 72 h of 40 μM BSO or co-treatment with 0.5 mM N-acetyl cysteine (NAC) or 0.5 mM GSH-Ethyl Ester (GSH-EE) following 5 days of GOT1 knockdown (*n* = 3 biological replicates), ***P = 0.0006, **P = 0.002, **P = 0.0087. **f** Subcutaneous xenograft growth of Pa-Tu-8902 iDox-shGOT1 cells treated with vehicle (black), 20 mg/kg BSO via drinking water (blue), doxycycline administered in the food (gray), or the combination (red), *P = 0.0193. **g** Relative abundance of gamma-glutamyl cysteine (γGC) (****P ≤ 0.0001 mock/BSO and ****P < 0.0001 dox/dox, BSO), GSH (****P ≤ 0.0001, ****P < 0.0001) GSSG (**P = 0.0041, ***P = 0.0003) and the GSH/GSSG ratio (***P = 0.0002, *P = 0.0469) from tumors in (**f**) (*n* = 8). Error bars represent mean ± SD or ± SEM (**f**, **g**). Two-tailed unpaired *T*-test or one-way ANOVA. Source data are provided as a Source Data file. x_c_^-^ system xc, γGC gamma-glutamyl cysteine, BSO buthionine sulfoximine, GSH, reduced glutathione, *GSSG* oxidized glutathione.

GSH can be synthesized de novo from cysteine or regenerated from oxidized glutathione (GSSG) via reduction by NADPH (Fig. 3a). GOT1 inhibition led to increased GSSG and NADP^+^, whereas the changes in GSH and NADPH varied among cell lines (Supplementary Fig. 5a, b). Thus, we hypothesized that inhibiting GSH biosynthesis may potentiate GOT1 knockdown.

The rate-limiting step in GSH synthesis can be inhibited by buthionine sulfoximine (BSO). GOT1 knockdown potently enhanced sensitivity to BSO after 24 h of drug treatment, contrasting a modest single agent response (Supplementary Fig. 6a). Moreover, exposure to BSO for 72 h augmented the potentiating effect (Fig. 3b, c and Supplementary Fig. 6b, c) while 6 h of treatment reduced GSH levels (Supplementary Fig. 6d), in line with previous kinetic data^11^. GOT1 inhibition potentiated the effect of BSO on proliferation (Fig. 3d, Supplementary Fig. 6e, and Supplementary Movies 1–4). The combinatorial effects on cell viability were rescued by supplementing exogenous GSH-EE or NAC (Fig. 3e and Supplementary Fig. 6f), suggesting a redox imbalance.

To determine whether this combination shows efficacy in vivo, we examined the effect of GOT1 and BSO in established xenograft tumors. Mice were engrafted with Pa-Tu-8902 iDox-shGOT1 cells and given dox via chow after 7 days. BSO was administered via drinking water on day 14. While no tumor regressions were observed, the combination of GOT1 and BSO significantly slowed tumor progression compared with single treatment arms (Fig. 3f). Knockdown was confirmed by immunoblot analysis (Supplementary Fig. 6g) and immunohistochemistry (Supplementary Fig. 6h). Ki67 staining and quantification (Supplementary Fig. 6i) illustrated a modest anti-proliferative effect in dox-treated arms.

We then measured glutathione species in tumor metabolite fractions to demonstrate the pharmacodynamics of BSO. BSO significantly reduced levels of gamma-glutamylcysteine, a product of GCL^12^, in BSO-treated tumors (Fig. 3g). We observed a significant reduction in GSH and GSSG (Fig. 3g), although the effect on GSH/GSSG was modest in double treatment tumors. These data demonstrate the on-target activity of BSO in established tumors, and that BSO-treated tumors are under redox stress. Together, our data reveal that PDA requires glutathione synthesis for viability and growth under GOT1 deficient conditions.

### GOT1 suppression potentiates ferroptosis sensitivity

**Fig. 4:**
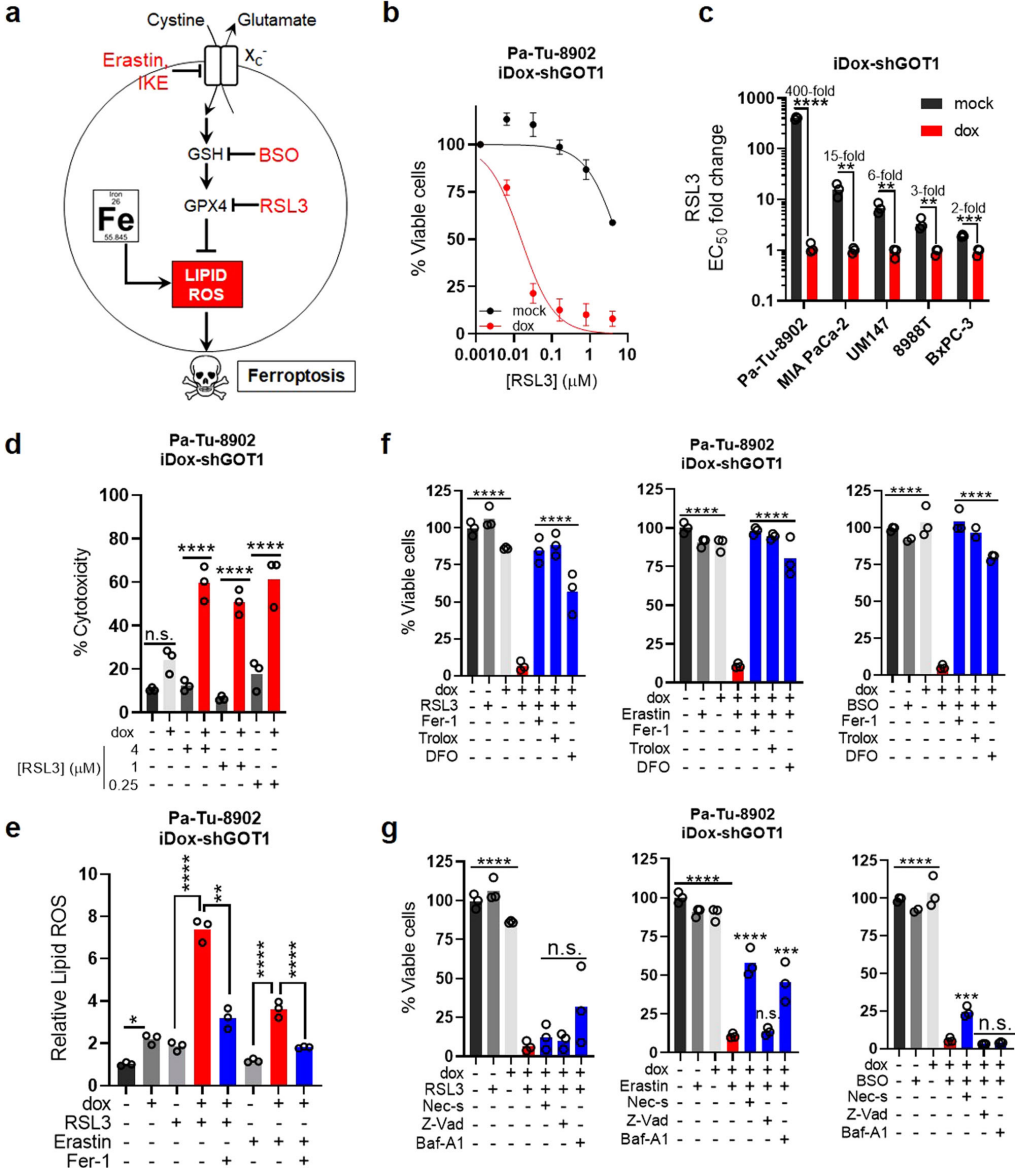
GOT1 inhibition potentiates ferroptosis. **a** Scheme of the GPX4 arm of ferroptosis. **b** Cell viability dose response curve at 24 h, *n* = 3 biological replicates. **c** EC_50_ fold changes in dose response following GOT1 knockdown and RSL3 treatment, *n* = 3 biological replicates, ****P ≤ 0.0001, **P = 0.005128, **P = 0.003314, **P = 0.008921, ***P = 0.000691. **d** % Cytotoxicity following GOT1 knockdown and RSL3 treatment at 24 h. Cytotoxicity was measured by LDH (Lactate dehydrogenase) release and normalized to a cell lysis control, *n* = 3 biological replicates, n.s. *P* = 0.1763, ****P = < 0.0001, ****P < 0.0001, ****P < 0.0001. **e** Relative lipid ROS in Pa-Tu-8902 iDox-shGOT1 treated with 32 nM RSL3 or 750 nM Erastin −/+ 1 μM Ferrostatin-1(Fer-1) for 6 h (*n* = 3 biological replicates), **P = 0.0057, ****P < 0.0001,****P < 0.0001,****P < 0.0001. **f** Cell viability of Pa-Tu-8902 iDox-shGOT1 cultured in vehicle (0.1% DMSO) −/+ dox (black and light gray), drug (32 nM RSL3, 750 nM Erastin, 40 μM BSO) −/+ dox (gray and red), or drug and dox (blue) in the presence of lipophilic antioxidants 1 μM Fer-1 and 100 μM Trolox, or an iron chelator 10 μM DFO (deferoxamine), ****P ≤ 0.0001 dox and dox/RSL3, Erastin, BSO; ****P < 0.0001 dox/RSL3 and dox/RSL3, Erastin, BSO/Fer-1, Trolox, DFO. Viability was assessed after 24 h of treatment for RSL3 and Erastin conditions and 72 h for BSO treatment conditions. GOT1 was knocked down for 5 days prior to treatment. Data are normalized to the –dox and vehicle-treated control (*n* = 3 biological replicates). **g** Cell viability following the procedure in (**f**) but in the presence of 10 μM Necrostatin-1 (Nec-s, necroptosis inhibitor), 50 μM ZVAD-FMK (Z-Vad, apoptosis inhibitor), or 1 nM bafilomycin A1 (BA-1, lysosomal acidification inhibitor), n = 3 biological replicates, ****P ≤ 0.0001 dox and dox/RSL3, Erastin, BSO; dox/Erastin, dox/Erastin/Nec-s, ****P  ≤ 0.0001; dox/Erastin, dox/Erastin/Baf-A1, ***P = 0.0002. dox/BSO, dox/BSO/Nec-s, ***P = 0.0021. Error bars represent mean ± SD. Two-tailed unpaired *T*-test or one-way ANOVA: Non-significant *P* > 0.05 (n.s., as noted). Source data are provided as a Source Data file. GPX4 glutathione peroxidase 4.

Previous work has demonstrated that some cell types are sensitive to erastin and BSO and that these drugs can kill cells by depleting GSH. The proximal effects of GSH depletion are mediated through loss of GPX4 activity, which utilizes GSH as a co-factor to detoxify lipid peroxides (Fig. 4a). This can lead to the lethal accumulation of lipid peroxides, and ferroptosis^13^. Ferroptosis is a form of oxidative, non-apoptotic, iron-dependent, cell death that is triggered by excessive lipid peroxide levels (Fig. 4a)^4,5^. While GOT1 inhibition does not induce ferroptosis, our data suggest it may prime PDA cells for ferroptosis.

To investigate whether GOT1 can sensitize PDA to ferroptosis, we first examined the combinatorial effect of GOT1 knockdown together with RSL3, a covalent inhibitor of GPX4 and direct inducer of ferroptosis^13^. RSL3 in combination with GOT1 knockdown was substantially more potent than as a single agent (Fig. 4b). GOT1 potentiated ferroptosis across a panel of PDA lines and this effect was independent of dox effects (Fig. 4c and Supplementary Fig. 7a, b). Moreover, the combination significantly reduced proliferation (Supplementary Fig. 7c) and potentiated cytotoxicity (Fig. 4d and Supplementary Fig. 7d). The GOT1 inhibition effect on cell number reflected both cytotoxic and growth inhibitory effects. This is indicated in Supplementary Movies 5–10 by a mixture of ferroptotic cells, noted by a cell blistering morphology^14^ and clonal outgrowth (Supplementary Movies 6 and 10). We then employed the C11-BODIPY lipid peroxidation sensor to investigate how inhibition of GPX4 and GOT1 affected lipid peroxidation. GOT1 inhibition alone led to modest lipid ROS, but potentiated lipid ROS accumulation when combined with RSL3 or erastin (Fig. 4e and Supplementary Fig. 7e, f). The potentiating effect could be reversed through co-treatment with the lipophilic antioxidant ferrostatin-1 (Fer-1) (Fig. 4e).

Next, we examined whether the GOT1 effect on ferroptosis could be prevented by co-treatment with agents that relieve lipid peroxidation or chelate iron^4,5^. Co-treatments with Fer-1 prevented the GOT1 potentiating effect across multiple small molecule treatments (Fig. 4f) and over a time course (Supplementary Fig. 7g). Moreover, treatment with the lipophilic antioxidant, Trolox, or iron chelator deferoxamine (DFO), provided significant protection (Fig. 4f). To rule out the possibility of apoptotic, necrotic, or autophagic cell death mechanisms, we co-treated GOT1 knockdown with well-characterized inhibitors of these cell death pathways. In line with ferroptotic cell death as the causative pathway, co-treatment with a pan-caspase inhibitor (Z-VAD-FMK), RIPK-1 inhibitor (Necrostatin-1), or lysosomal acidification inhibitor (Bafilomycin A1) offered limited protection, compared with lipophilic antioxidants or iron chelation (Fig. 4g and Supplementary Fig. 7h). Overall, our data demonstrate GOT1 inhibition primes PDA for ferroptosis in cell culture.

### Mitochondrial inhibition potentiates ferroptosis

The canonical biochemical role of GOT1 is to support mitochondrial function through the malate-aspartate shuttle (Fig. 1a). In line with this, we observed that GOT1 inhibition for 5 days reduced NAD^+^ and increased NADH levels (Supplementary Fig. 8a). Concurrently, we observed an increased AMP/ATP ratio, an additional indicator of energetic stress (Supplementary Fig. 8b). Thus, we hypothesized that GOT1 inhibition was impairing mitochondrial metabolism. We conducted oxygen consumption rate (OCR) measurements to obtain a physiological readout of mitochondrial respiration and found GOT1 knockdown reduced basal OCR levels (Supplementary Fig. 8c, d). To determine the component role of mitochondrial inhibition on the GOT1 inhibition ferroptotic potentiating effect, we tested if mitochondrial poisons could directly enhance the activity of ferroptotic stimuli. Indeed, we found that inhibition of Complex V or I, with oligomycin or phenformin, respectively, potentiated ferroptosis when combined with GPX4 inhibition (Supplementary Fig. 8e, f). These data corroborate those from a recently published genetic modifier screen in which the authors demonstrate that mitochondrial dysfunction and GPX4 inhibition are synthetic lethal^15^. Our data suggest a similar mechanism may be operative in pancreatic cancer cells.

### GOT1 inhibition primes PDA for ferroptosis by promoting labile iron

**Fig. 5:**
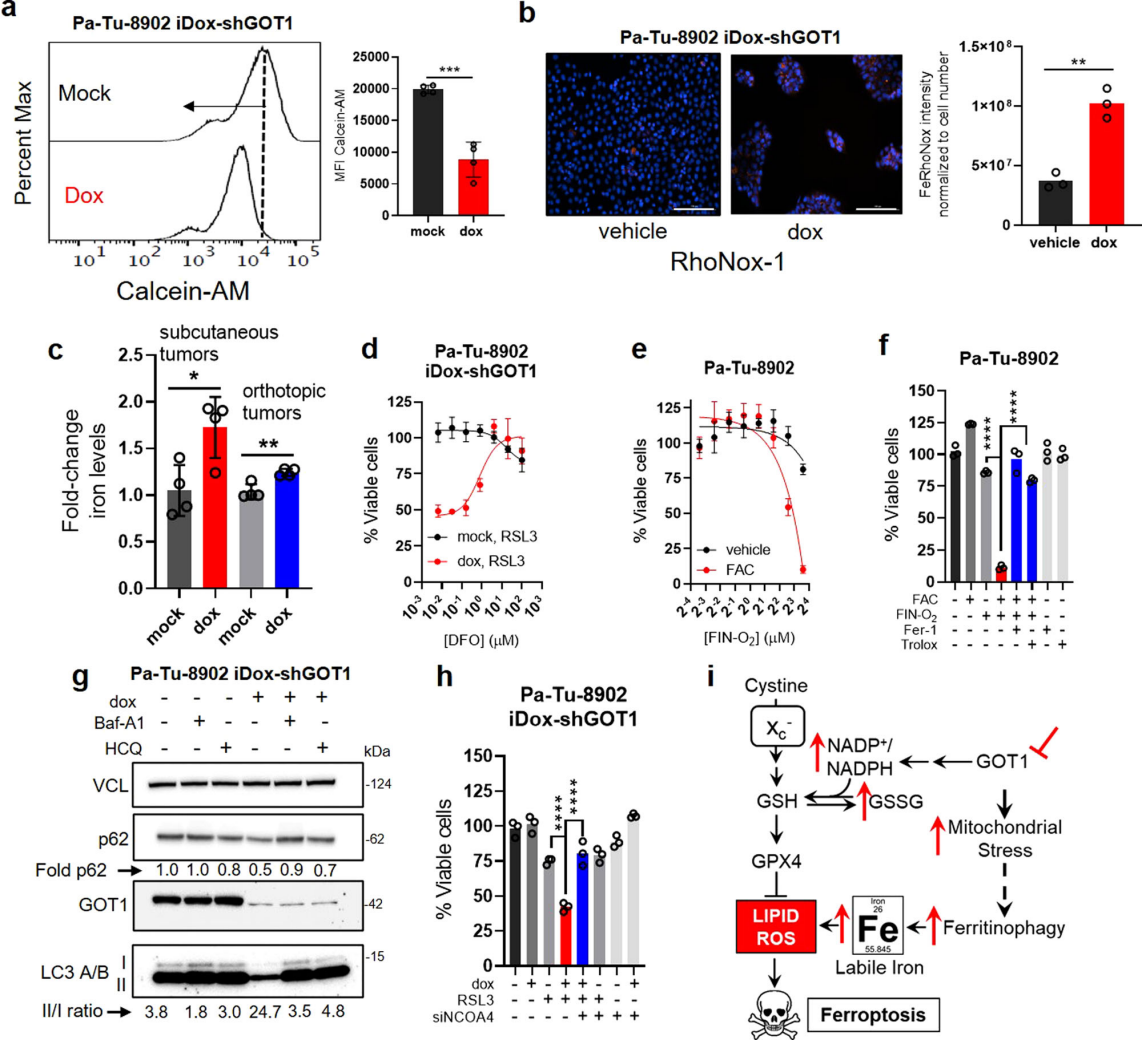
GOT1 inhibition promotes labile iron release. **a** Calcein-AM histogram (**a**) and mean fluorescence intensity (MFI) (***P = 0.0002) upon 5 days of GOT1 knockdown, *n* = 3 biological replicates. **b** Visualization and quantification of GOT1 knockdown cells treated with the iron probe RhoNox-1, *n* = 3 biological replicates. **P = 0.0013. Scale bars represent 200 μm. Image is representative of two independent experiments. **c** ICP-MS (Inductively coupled plasma mass spectrometry) measurements of iron in subcutaneous and orthotopic tumors following GOT1 knockdown, *n* = 4 tumors, *P = 0.0191 and **P = 0.0036. **d** Cell viability normalized to vehicle of cells co-treated with 32 nM RSL3 and increasing doses of DFO, *n* = 2 biological replicates. **e** Cell viability dose response to FINO_2_ with or without 200 µM ferric ammonium citrate (FAC), *n* = 3 biological replicates. Cell viability of FINO_2_ with 200 µM FAC (ferric ammonium citrate) (**f**) or the indicated rescue conditions *n* = 3 biological replicates, ****P = < 0.0001. **g** Western of autophagy markers following GOT1 knockdown representative of two independent experiments. **h** Cell viability in cells co-treated with RSL3 and siNCOA4 following GOT1 knockdown, *n* = 3 biological replicates, ****P ≤ 0.0001. **i** Model describing the GOT1-mediated potentiation of ferroptosis through ferritinophagic iron release. Error bars represent mean ± SD. Two-tailed unpaired *T*-test or one-way ANOVA. Source data are provided as a Source Data file.

Recent studies have demonstrated that metabolic disturbances lead to the adaptive release of iron from intracellular stores to support energy metabolism in the mitochondria^16–19^. Given that susceptibility to ferroptosis is linked to the availability of free iron^20,21^, we hypothesized that intracellular iron levels may be increased in response to GOT1 inhibition-mediated energetic stress. We tested this hypothesis first by measuring Calcein-AM, a fluorescein-derived probe that is quenched when bound to ferrous iron (Fe^2+^)^22^. Calcein-AM staining of GOT1 proficient cells defined baseline fluorescence. GOT1 knockdown cells shifted fluorescence distribution to lower intensity, indicating labile iron pools were increased following GOT1 knockdown (Fig. 5a and Supplementary Fig. 9a). This observation was corroborated employing the RhoNox-1 (ref. ^23^) iron probe in cultured cells (Fig. 5b) and the observation that total iron levels were elevated in subcutaneous and orthotopic tumors (Fig. 5c). The effect of labile iron was evident phenotypically, where higher concentrations of the iron chelator DFO were required to rescue cell viability under GOT1-deficient conditions (Fig. 5d and Supplementary Fig. 9b). GOT1 inhibition augmented sensitivity to FINO_2_, a pharmacological iron oxidant^24^ (Supplementary Fig. 9c). Finally, supplementation with ferric ammonium citrate (FAC) potentiated ferroptosis (Fig. 5e, f and Supplementary Fig. 9d). Taken together, these data demonstrate that GOT1 knockdown promotes labile iron and iron potentiates ferroptosis.

Iron levels can be altered by downregulating iron efflux, upregulating iron uptake, or promoting the degradation of intracellular iron carriers; i.e., ferritin (FTN) or heme^25^ (Supplementary Fig. 9e). To test which of these pathways was leading to increased intracellular iron, we first examined expression of iron transport proteins. GOT1 knockdown did not significantly alter expression of the iron export protein *SLC40A1* (Supplementary Fig. 9f). Expression of heme oxygenase 1 (*HMOX1*), which releases labile iron through the degradation of heme, was unaltered in Pa-Tu-8902 but was upregulated in MIA PaCa-2 (Supplementary Fig. 9f). GOT1 knockdown had a minimal effect on the iron-responsive proteins Nuclear Receptor Coactivator 4 (NCOA4) and Transferrin Receptor 1. By contrast, Iron Responsive Element-Binding Protein 2 (IRP2) levels were lower and FTN levels were elevated, both of which would be expected under higher iron loads (Supplementary Fig. 9g). Transcriptome profiling and gene set enrichment analysis in Pa-Tu-8902 cells revealed evidence for the catabolic pathway signatures “Lysosome” and “Autophagy-Lysosome”^26^ (Supplementary Fig. 10a). In line with these observations, GOT1 knockdown increased the LC3-A/B II/I ratio and reduced P62 levels, consistent with increased autophagic flux (Fig. 5g and Supplementary Fig. 10b). Lysosomal inhibitors hydroxychloroquine and Baf-A1 were used to illustrate that GOT1 inhibition increased flux, as opposed to stalling the autophagic process.

Labile iron release from intracellular protein stores requires their acidification and degradation in either the lysosome or endosome, mediated by vacuolar (V-)ATPases^27–29^. We found that blocking V-ATPases prevented GOT1-mediated ferroptosis (Supplementary Fig. 10c) across several conditions. Intracellular iron levels can be regulated by NCOA4-dependent autophagic process termed ferritinophagy^27^. NCOA4 is an autophagosome cargo receptor that binds to the ferritin heavy chain sequestering ferritin for degradation by the autolysosome to release labile iron. Given the impact of GOT1 knockdown on the induction of autophagy wondered if inhibiting ferritinophagy via NCOA4 ablation would prevent GOT1-mediated ferroptosis. NCOA4 knockdown by siRNA reversed the potentiation of ferroptosis in Pa-Tu-8902 cells (Fig. 5h and Supplementary Fig. 10d, e), supporting the model that GOT1 promotes labile iron through ferritinophagy in this cell line (Fig. 5i).

## Discussion

We report that inhibition of GOT1 suppresses the growth of numerous PDA cell lines, primary cancer models, and xenograft tumors, while rendering some PDA cells susceptible to ferroptosis. Ferroptosis could be triggered by inhibiting cystine import, GSH synthesis, or GPX4 in synergy with GOT1. This effect is likely due to a combination of redox disruption, mitochondrial inhibition, and adaptive labile iron release, all of which are known to potentiate ferroptosis (Fig. 5i).

The dependency on exogenous cystine in GOT1 knockdown cells was identified using a synthetic lethal chemical screening strategy. We then demonstrated that exogenous cystine can be selectively exploited in GOT1 knockdown cells and tumors. This metabolic rewiring could be exploited by dietary means, which has a major influence on the nutrient composition within pancreatic tumors^10^. This result adds to a body of literature indicating how the metabolic environment can influence sensitivity to therapy.

The inhibition of de novo GSH biosynthesis with BSO also potentiated tumor inhibition by GOT1. Previous efforts with BSO have indicated that many tumor types employ compensatory mechanisms to tolerate glutathione inhibition^11^. Indeed, we too have observed that inhibition of de novo glutathione biosynthesis is not sufficient to induce ferroptosis in PDA^14^. This points to logical combinations to enhance therapeutic efficacy of targeting GSH metabolism. To this end, we and others engaged in drug discovery campaigns to develop small molecule inhibitors of GOT1^8,30–32^. However, future studies are yet required to improve the properties of candidate GOT1 inhibitors for in vivo studies.

Labile iron release could support mitochondrial metabolism and represent a targetable vulnerability in cancer. Several groups have shown iron released from lysosomes or endosomes supports mitochondrial function^16–19^ and that organelle proximity and communication could mediate this process^33^. We demonstrate that GOT1 inhibition represses mitochondrial metabolism and that inhibiting mitochondrial metabolism potentiates ferroptosis. Blocking V-ATPases prevented GOT1-mediated ferroptosis in two PDA cell lines. Iron released from lysosomes or endosomes may support mitochondrial function when suppressed by the inhibition of the malate-aspartate shuttle or mitochondrial inhibition. GPX4 is induced during the PGC-1α transcriptional program for mitochondrial biogenesis^34^, suggesting a relationship where OXPHOS activity is met with an increased need for GPX4^15,35^. GPX4 deletion or pharmacological inhibition is synthetic lethal with oligomycin where mitochondrial GPX4 expression rescues this effect indicating that the mitochondria are a vital lipid peroxidation site^15^. This relationship seems operative in humans, as patients harboring mitochondrial defects showed higher GPX4 protein levels^36^. Future work is needed to demonstrate a direct link between labile iron in relation to mitochondrial function and support from lysosomal and endosomal sources of labile iron.

Not all KRAS mutant cell lines are dependent on GOT1. We found that some PDA cell lines are highly resistant to GOT1 inhibition and seemingly bypass GOT1 for redox balance. Resistant PDA cell lines could bypass GOT1 through reductive carboxylation^6^, which has been shown to support growth in cancer cells with defective mitochondria^37,38^ and support redox homeostasis^39^. The differences in glutamine flux could account for heterogeneous sensitivity GOT1 inhibition.

The role of GOT1 in ferroptosis has been the subject of previous study in several other tumor types. Our data lie in contrast to some previous work, which have suggested that GOT1 inhibition protects cells from ferroptosis by blocking mitochondrial metabolism or glutaminolysis^4,35,40,41^. By contrast, we reveal iron release following GOT1 knockdown can promote ferroptosis, which is in line with other previous studies^20,21^. Indeed, a growing wealth of work indicates that the genotype^42^, nutrient environment^43^, tissue of origin^13,44^, and cell-autonomous metabolism^20,45^ are drivers of ferroptosis sensitivity. The differences emerging from these studies likely reflect the incomplete understanding of how these various factors dictate sensitivity to ferroptosis. Thus, our study provides clarity regarding the metabolic regulation of ferroptosis in PDA.

Finally, despite the profound phenotypes observed in cell culture settings, our efforts to engage ferroptosis in tumor models in vivo led to modest results without robust evidence for ferroptosis. Similar results have been observed in the field^13,45–47^, and suggest an in vivo microenvironmental component could provide resistance to ferroptosis. Indeed, fibroblasts have been shown to release GSH and cysteine^48^, while more recently, circulating mono-unsaturated fatty acids have been shown to prevent ferroptosis of metastatic cells in vivo^49^. That said, we previously provided the first evidence of ferroptosis in an autochthonous PDA model following systemic genetic depletion of *Slc7a11*^14^, and others have shown more robust tumor growth suppression targeting ferroptosis-sensitive cell states^42,44^. Uncovering which environmental, genetic, and metabolic contexts influence ferroptosis susceptibility will be critical for translating this strategy for cancer therapy.

## Methods

### Cell culture

PL45, Capan-1, BxPC-3, MIA PaCa-2, Panc10.05, Panc03.27, PANC-1, Capan-2, HPNE, IMR-90 were obtained from ATCC. Pa-Tu-8902, Pa-Tu-8988T, YAPC, and Hup T3 were obtained from DSMZ. hPSC was a generous gift from Rosa Hwang^50^. The UM PDA primary cell cultures (UM147, UM5, UM90, and UM19) were obtained from surgically resected samples and established through murine xenograft^7^. All commercial cell lines and UM PDA primary cultures were validated by STR profiling and tested negative for mycoplasma infection (Lonza, LT07-701). Cells were maintained under standard conditions at 37 °C and 5% CO_2_. Cells were grown either in regular DMEM (GIBCO, #11965) or RPMI (GIBCO, #11875) or in DMEM (GIBCO, #21013024) or RPMI (GIBCO, A1049101) without cystine supplemented with 10% FBS (Corning, 35-010-CV), unless otherwise indicated. Cultures involving inducible sh-mediated knockdown were supplemented with doxycycline-hyclate (Dox) at 1 µg/mL (Sigma, D9891) for 5 days prior to experiments.

### Lentiviral-mediated shRNA transduction

Parental PDA cell lines were transduced with lentivirus containing shRNA plasmids at optimized viral titers. Stable cell lines were established post-puromycin or blasticidin selection. The Tet-pLKO-puro entry vector (A gift from Dmitri Wiederschain, Addgene, 21915) was used to establish dox-inducible shRNA targeting GOT1 (ref. ^6^).

### CRISPR construct knockout GOT1 in PDA cells

PDA cell lines Pa-Tu-8902, MIA PaCa-2, and Capan-1 were either transfected with plentiCRISPR-sgGOT1 (A gift from David Sabatini, Addgene 72874) using Lipofectamine 3000 (Invitrogen) or viral transduction using lentiviral particles of plentiCRISPR-sgGOT1 produced in 293FT cells (Thermo Fisher) by FuGENE 6 Tranfection Reagent (Promega) and lentiviral packaging plasmid psPAX2 (Addgene, 12260) and VSV-G envelope expressing plasmid pMD2.G (Addgene,12259) (Gifts from Didier Trono). Puromycin selected and pooled stable cells were then subject to cellular assays and western blot analysis.

### Clonogenic assays

Cells were plated in six-well plates in biological triplicates at 300–600 cells per well in 2 mL of media. Dox-media were changed every 2 days. Assays were concluded after 10–15 days by fixing in −20 °C cold 100% methanol 10 min and staining with 0.5% crystal violet 20% methanol solution for 15 min. Colonies were quantified using ImageJ or manually counted.

### Cell proliferation assays

Cells were seeded in a 96-well plate at 1000 cells per well in 0.1 mL of media. Indicated treatments were applied the subsequent day. Media was changed every 2 days. At the indicated time points, media was aspirated and plates were frozen. One hundred microliters of CyQUANT (Invitrogen, C7026) was added to each well for measurements, or 10 µL of WST-1 reagent was added directly to the culture media (Sigma, #11644807001). Relative proliferation was determined by the fluorescence intensity at 530 nm for CyQUANT or 450 nm for WST-1 using a SpectraMax M3 plate reader.

### Cell viability assays

Cells were plated in a 96- or 384-well plate format at 1000 cells per well. Cells were seeded overnight, then treated with compounds at indicated concentrations and for indicated lengths of time. All viability assays utilized the Cell-Titer-Glo 2.0 reagent (Promega, G9243) according to the manufacturer’s instructions. Media was aspirated followed by the addition of 100 µL of Cell-Titer-Glo 2.0 reagent to each experimental well. Plates were gently agitated for 10 min to promote adequate mixing. Luminescence was subsequently measured using a SpectraMax M3 plate reader.

### Cytotoxicity assays

Cytotoxicity assays were performed using the CytoTox96 kit (Promega, G1780) that quantifies LDH release. Cell numbers were first optimized utilizing a cell lysis solution following the manufacturer’s protocol. 15,000–20,000 Pa-Tu-8902 or Mia PaCa-2 iDox-shGOT1 cells were cultured in dox and seeded per well one day prior to drug treatment. Cells were exposed to drug for 24 h followed by media collection. Maximum LDH release controls were obtained by treating cells with a cell lysis solution (Promega, G182A) for 30 min prior to reagent incubation. Assay reagents were administered to each well and incubated at room temperature for 30 min. Absorbance was measured using a SpectraMax M3 plate reader. % Cytotoxicity was obtained by normalizing LDH release counts to that of the cell lysis treatment.

### RNAi

On-TARGET siRNA smart pools targeting NCOA4 (Dharmacon) were transfected into Pa-Tu-8902 or Mia PaCa-2 iDox-shGOT1 cells previously seeded in 96-well plates using Lipofectamine RNAiMAX (Thermo Fisher) per the manufacturer’s instructions. MEM-media (Thermo Fisher) was used as a mock treatment.

### Quantitative RT-PCR

Total RNA was extracted using the RNeasy Mini Kit (Qiagen, 74104) and reverse transcription was performed from 2 µg of total RNA using the iScript cDNA synthesis kit (BioRad, 1708890), according to the manufacturer’s instructions. Quantitative RT-PCR was performed with Power SYBR Green dye (Thermo, 4367659) using a QuantStudio 3 System (Thermo). PCR reactions were performed in triplicate and the relative amount of cDNA was calculated by the comparative *C*_T_ method using *RPS21* as an endogenous control. RT-PCR was performed in at least three biological replicates. Primer sequences are provided in Supplementary Table 1.

### Detection of reactive oxygen and labile iron by flow cytometry

Cells were plated in six-well plates 2 days before incubation with indicated treatments. Cells were then washed twice with 1× PBS, and stained for 20–30 minutes with 2 µM C11-BODIPY (Invitrogen, D3861) or for 10 min with 0.2 µM Calcein-AM (Invitrogen, C1430) in phenol red-free DMEM. Cells were co-stained with Sytox-blue (Invitrogen, S34857) to account for cell viability. Following staining, cells were washed twice with PBS, trypsinized (0.25%, Life Technologies, 25200-056), and naturalized with pure FBS at a 1:1 volume. Cells were then collected in 50 µL PBS, and moved to round-bottom 96-well plates, on ice, for measurements. A minimum of 8000 cells were analyzed per condition. C11-BODIPY and Calcein-AM signals were analyzed in the FITC channel, while Sytox-blue was analyzed in the DAPI channel on a ZE5 Cell analyzer (Bio-Rad). Analysis of data was performed using FlowJo v.10 software. Relative labile iron levels were calculated based on the ratio of Calcein-AM mean fluorescence intensity of control vs. dox-treated samples. Gating parameters are presented in Supplementary Fig. 11.

### Image-based detection of labile iron

GOT1 was knocked down for 5 days in Pa-Tu-8902 iDox-shGOT1 and seeded at 10,000 cells per well. Cells were treated the following day with Hoechst (1 μg/mL final concentration) and RhoNox-1 (Goryo, GC901) at 500 nM for 6 h then imaged using a Cytation5 Cell Imaging Multi-Mode Reader (BioTek, VT, USA). Hoechst was imaged using a 365 nm LED in combination with an EX 377/50 EM 447/60 filter cube. RhoNox-1 was imaged using a 523 nm LED in combination with an EX 531/40 EM 593/40 filter cube. Image analysis was completed using Gen5 software (BioTek).

### Trace element analysis

Tissue samples were analyzed for metals by inductively coupled plasma mass spectrometry (Perkin Elmer Nexion 2000). The internal standard was 50 ppb Bismuth. Briefly, the tissues were digested with 2 mL/g total wet weight nitric acid (Trace metal grade; Fisher) for 24 h, and then digested with 1 mL/g total wet weight hydrogen peroxide (Trace metal grade; Fisher) for 24 h at room temperature. The samples were preserved at 4 °C until quantification of metals. Ultrapure water (VWR Chemicals ARISTAR^®^ULTRA) was used for final sample dilution.

### Xenograft studies

Animal experiments were conducted in compliance with ethical regulations approved by the Office of Laboratory Animal Welfare and the Institutional Animal Care and Use Committees (IACUC) of the University of Michigan (Protocol#: PRO00008877). NOD scid gamma (NSG) mice (Jackson Laboratory, 005557), 6–8 or 8–10 weeks old of both sexes, were maintained in the facilities of the Unit for Laboratory Animal Medicine under specific pathogen-free conditions. Mice were maintained in conventional cages at room temperatures of 72 °F (±4 °F), humidity ranging from 30 to 70%, and a 12-h light cycle. Mice had access to chow and water ad libitum. Stable PDA cell lines containing a dox- inducible shRNA against GOT1 were trypsinzied and suspended at 1:1 ratio in DMEM (Gibco, 11965-092) cell suspension to Matrigel (Corning, 354234). 150–200 μL were used per injection. For subcutaneous xenograft studies, 0.5 × 10^6^ cells were implanted into the lower flanks. Doxycycline (dox) chow (BioServ, F3949) was fed to the +dox groups. Orthotopic tumors were established by injecting 5 × 10^4^ Pa-Tu-8902 iDox-shGOT1 #1 pFUGW-FLuc into 8–10-week-old NSG mice. Cysteine-free chow (LabDiet) was customized from Baker Amino Acid (LabDiet, 5CC7) to remove cysteine and balance protein levels with increased valine and aspartic acid. BSO was delivered in the drinking water at 20 mM. All treatments began on day 7 after implantation.

Subcutaneous tumor size was measured with digital calipers at the indicated endpoints. Tumor volume (*V*) was calculated as *V* = 1/2(length x width^2^). Bioluminescence (BLI) of orthotopic tumors were measured via IVIS SpectrumCT (PerkinElmer) following an intraperitoneal injection of 100 μL beetle luciferin (40 mg/mL in PBS stock) (Promega, E1605). BLI was analyzed with Living Image software (PerkinElmer). At endpoint, final tumor volume and mass were measured prior to processing. Tissue was either fixed in zinc formalin fixative (Z-fix, Anatech LTD, #174) for >24 h for histological and/or histochemical analysis, or snap-frozen in liquid nitrogen then stored at −80 °C until metabolite or protein analysis.

### Western blot analysis

Stable iDox-shNT and iDox-shGOT1 cells were cultured with or without dox media and protein lysates were collected after 5 days using RIPA buffer (Sigma, R0278) containing protease inhibitor cocktail (Sigma/Roche, 04 693 132 001). Samples were quantified with Pierce BCA Protein Assay Kit (Thermo Fisher, 23225). Ten to 40 µg of protein per sample were resolved on NuPAGE Bis-Tris Gels (Invitrogen, NP0336) and transferred to an Immobilon-FL PVDF membrane (Millipore, IPVH00010). Membranes were blocked in 5% non-fat dry milk in distilled H_2_O prior to incubation with the primary antibody. The membranes were washed with TBS-Tween followed by a 1 h exposure to the appropriate horseradish peroxidase-conjugated secondary antibody. The membranes were washed in de-ionized water for 15–30 min then visualized using a Bio-Rad ChemiDox MP Imaging System (Bio-Rad, 17001402).

The following human antibodies were used for immunoblot: 1:1000 dilution aspartate aminotransferase (Abcam, ab171939, clone EPR12144(B) and ab170950, clone EPR12145), 1:1,000 dilution Rabbit LC3 A/B (CST, 12741, clone D3U4C), 1:1,000 dilution Rabbit NCOA4 (ARA70) (Bethyl Laboratories, A302-272A, Polyclonal), 1:1,000 dilution Rabbit IRP2 (CST, 37135S, clone D6E6W), 1:1000 dilution Ferritin (Abcam, ab75973, clone EPR3004Y), 1:1,000 dilution Transferrin Receptor (Abcam, ab84036, polyclonal), 1:1,000 p62 (Abcam, ab109012, clone EPR4844). The following loading controls were used at a 1:10,000 dilution: Vinculin (CST, 13901, clone E1E9V), HSP-90 (CST, 4877S, clone C45G5), Rabbit β-Actin (CST, 4970 L, clone 13E5) or GAPDH (CST, 2118, clone 14C10).

### Histology

Mice were sacrificed by CO_2_ asphyxiation followed by tissue harvesting and fixation overnight at room temperature with Z-fix solution (Z-fix, Anatech LTD, #174). Tissues were the processed by using a Leica ASP300S Tissue Processor, paraffin embedded, and cut into 5-μm sections. Immunohistochemistry was performed on Discovery Ultra XT autostainer (Ventana Medical Systems Inc.) and counterstained with hematoxylin. IHC slides were scanned on a Panoramic SCANslide scanner (Perkin Elmer), and then annotation regions encompassing greater than 1 mm of tissue were processed using Halo software (Indica Labs).The following antibodies were used for IHC: GOT1 (AbCam, ab171939), Ki-67 (CST, 9027), Cleaved Caspase-3 (CST, 9664).

### Metabolomics

Cells were plated at 500,000 cells per well in six-well plates or ~1.5 million cells per 10-cm dish. At the endpoint, cells were lysed with dry-ice cold 80% methanol and extracts were then centrifuged at 10,000 × *g* for 10 min at 4 °C and the supernatant was stored at −80 °C until further analyses. Protein concentration was determined by processing a parallel well/dish for each sample and used to normalize metabolite fractions across samples. Based on protein concentrations, aliquots of the supernatants were transferred to a fresh microcentrifuge tube and lyophilized using a SpeedVac concentrator. Dried metabolite pellets were re-suspended in 45 μL 50:50 methanol:water mixture for LC–MS analysis. Data were collected using previously published parameters^51,52^.

The QqQ data were pre-processed with Agilent MassHunter Workstation Quantitative Analysis Software (B0700). Each sample was normalized by the total intensity of all metabolites to scale for loading. Finally, each metabolite abundance level in each sample was divided by the median of all abundance levels across all samples for proper comparisons, statistical analyses, and visualizations among metabolites. The statistical significance test was done by a two-tailed *t*-test with a significance threshold level of 0.05.

### Seahorse mito stress test

MiaPaCa-2 cells were seeded at 2 × 10^4^ cells/well in 80 μl/well of normal growth media (DMEM with 25 mM Glucose and 2 mM Glutamine) in an Agilent XF96 V3 PS Cell Culture Microplate (#101085-004). To achieve an even distribution of cells within wells, plates were incubated on the bench top at room temperature for 1 h before incubating at 37 °C, 5% CO_2_ overnight. To hydrate the XF96 FluxPak (#102416-100), 200 μL/well of sterile water was added and the entire cartridge was incubated at 37 °C, no CO_2_ overnight. The following day, 1 h prior to running the assay, 60 μL/well of growth media was removed from the cell culture plate, and cells were washed twice with 200 μL/well of assay medium (XF DMEM Base Medium, pH 7.4 (#103575-100) containing 25 mM glucose (#103577-100) and 2 mM glutamine (#103579-100)). After washing, 160 μL/well of assay medium was added to the cell culture plate for a final volume of 180 μL/well. Cells were then incubated at 37 °C, without CO_2_ until analysis. One hour prior to the assay, water from the FluxPak hydration was exchanged for 200 μL/well of XF Calibrant (#100840-000) and the cartridge was returned at 37 °C, without CO_2_ until analysis. Oligomycin (100 μM), FCCP (100 μM), and Rotenone/Antimycin (50 μM) from the XF Cell Mito Stress Test Kit (#103015-100) were re-constituted in assay medium to make the indicated stock concentrations. Twenty microliters of Oligomycin was loaded into Port A for each well of the FluxPak, 22 μL of FCCP into Port B, and 25 μL of Rotenone/Antimycin into Port C. Port D was left empty. The final FCCP concentration was optimized to achieve maximal respiration in each condition.

The Mito Stress Test was conducted on an XF96 Extracellular Flux Analyzer and OCR was analyzed using Wave 2.6 software. Following the assay, OCR was normalized to cell number with the CyQUANT NF Cell Proliferation Assay (C35006) from Thermo Fisher according to manufacturer’s instructions.

### RNA-seq

Pa-Tu-8902 and Mia PaCa-2 iDox-shGOT1 cells were collected in lysis buffer following 5 days of GOT1 knockdown. Total RNA was extracted using the RNeasy Mini Kit (Qiagen, 74104) according to the manufacturer’s instructions, and RNA with RIN of at least 7 were used to prepare libraries for sequencing. Strand-specific, poly-A + libraries were produced using NEBNext Ultra II Directional RNA Library Prep Kit (New England Biolab, E7760L), the Poly(A) mRNA Magnetic Isolation Module (New England Biolab, E7490L), and NEBNext Multiplex Oligos for Illumina Unique Dual (New England Biolab, E6440L). The quality and quantity of cDNA libraries were assessed by TapeStation (Agilent) and qPCR using Kapa’s library quantification kit for Illumina Sequencing platforms (Kapa Biosystems, KK4835). Sequencing was performed on the NovaSeq-6000 (Illumina), yielding 150-base, paired-end reads. Bcl2fastq2 (Illumina) was used to demultiplex and generate fastq files.

Reads were trimmed using Trimmomatic v0.39^53^ with parameters ILLUMINACLIP: TruSeq3-PE.fa:2:30:10:2:true LEADING:3 TRAILING:3 MINLEN:35 SLIDINGWINDOW:4:15. Library qualities were determined using FastqQC.0.11.8 for trimmed reads (available online at: http://www.bioinformatics.babraham.ac.uk/projects/fastqc/). Paired-end alignments and gene-level counts were obtained using RSEM v1.3.1^54^. STAR v2.5.2a was the aligner called by RSEM and the default parameters were used. Principal component analysis of gene expression (FPKM, log10-transformed) was used to assess the similarity of samples and determine the major sources of variance.

Differential gene expression analysis was performed using DESeq2 v1.26.0^55^ and the default shrinkage estimator was used to compute log_2_ fold changes. The differentially expressed and overlapping genes were selected by padj < 0.01 (Fisher’s Exact Test) and fold change 1.5 (up or down). The reference sequence hg38 (GRCh38) and annotations, including gene IDs, were obtained from GENCODE v29^56^. Differentially expressed genes were analyzed using GSEA^57^ using KEGG gene categories and an autophagy-lysosome signature^29^. The full data are publicly available at GEO (GSE157830).

### Malate-aspartate shuttle expression analysis

Gene expression data show in Supplementary Fig. 1d were obtained from the Cancer Cell Line Encyclopedia (https://portals.broadinstitute.org/ccle/).

### Incucyte imaging

One thousand cells per well were plated in clear flat-bottom 96-well plates and treated with the mock, dox, erastin, BSO, RSL3, or FINdO_2_ with or without dox. Cell images were taken every 6 h for 72 h using the image lock and brightfield setting with an Incucyte^®^ S3 Live-Cell Analysis System (Sartorius). The default cell masking application was applied to delineate cell boundaries.

### Statistical analysis

Statistics were performed using GraphPad Prism 7 (Graph Pad Software Inc). Groups of 2 were analyzed using the unpaired two-tailed Student’s *t* test and comparisons across more than 2 groups were conducted using the one-way ANOVA Tukey post-hoc test. All error bars represent mean with standard deviation, unless noted otherwise. A *P* value of less than 0.05 was considered statistically significant. All group numbers and explanation of significant values are presented within the figure legends.

### Reporting summary

Further information on research design is available in the Nature Research Reporting Summary linked to this article.

## Supplementary information

Supplementary Information

Peer Review File

Description of Additional Supplementary Files

Supplemental Movies 1-10

Reporting Summary

## Data Availability

The RNA sequencing raw data files have been deposited at GEO under the accession code: GSE157830. The metabolomics data and other data supporting this study are available within the Source Data File. All other data is available in the Article and Supplementary Information. Source data are provided with this paper.

## Source data

Source Data
