# Supplementary material for: GOT1 inhibition promotes pancreatic cancer cell death by ferroptosis
Source: Nat Commun. 2021 Aug 11;12:4860. doi: 10.1038/s41467-021-24859-2 (PMC8357841; doi:10.1038/s41467-021-24859-2)
Supplement: Supplementary file 1 — Supplementary Information [file 41467_2021_24859_MOESM1_ESM.pdf]

# GOT1 Inhibition promotes Pancreatic Cancer cell death by Ferroptosis

Daniel M. Kremer[1,2], Barbara S. Nelson[3], Lin Lin[1], Emily L.Yarosz[4], Christopher J. Halbrook[1], Samuel A. Kerk[3], Peter Sajjakulnukit[3], Amy Myers[1], Galloway Thurston[1], Sean W. Hou[1], Eileen S. Carpenter[5], Anthony C. Andren[1], Zeribe C. Nwosu[1], Nicholas Cusmano[1], Stephanie Wisner[1], Nneka E. Mbah[1], Mengrou Shan[1], Nupur K. Das[1], Brian Magnuson[6,7], Andrew C. Little[7], Milan R. Savani[8], Johanna Ramos[1], Tina Gao[1], Stephen A. Sastra[9,10], Carmine F. Palermo[9,10], Michael A. Badgley[9-11], Li Zhang[1], John M. Asara[12,13], Samuel K. McBrayer[14], Marina Pasca di Magliano[7,15,16], Howard C. Crawford[1,7], Yatrik M. Shah[1,5,7], Kenneth P. Olive[9,10], Costas A. Lyssiotis[1,5,7*]

[1]Department of Molecular & Integrative Physiology, University of Michigan, Ann Arbor, MI 48109, USA
[2]Graduate Program in Chemical Biology, University of Michigan, Ann Arbor, MI 48109, USA
[3]Graduate Program in Cancer Biology, University of Michigan, Ann Arbor, MI 48109, USA
[4]Immunology Graduate Program, University of Michigan, Ann Arbor, MI 48109, USA
[5]Department of Internal Medicine, Division of Gastroenterology and Hepatology, University of Michigan, Ann Arbor, MI 48109, USA
[6]Department of Biostatistics, School of Public Health, University of Michigan, Ann Arbor, MI.
[7]Rogel Cancer Center, University of Michigan, Ann Arbor, MI 48109, USA
[8]Medical Scientist Training Program, University of Texas Southwestern Medical Center, Dallas, TX 75390, USA
[9]Division of Digestive and Liver Diseases, Department of Medicine, Columbia University Medical Center, New York, NY 10032, USA
[10]Herbert Irving Comprehensive Cancer Center, Columbia University Medical Center, New York, NY 10032, USA
[11]Department of Pathology, Columbia University Medical Center, New York, NY 10032, USA
[12]Division of Signal Transduction and Mass Spectrometry Core, Beth Israel Deaconess Medical Center, Boston, MA, USA 02115
[13]Department of Medicine, Harvard Medical School, Boston, MA, USA 02115
[14]Children's Medical Center Research Institute and Department of Pediatrics, University of Texas Southwestern Medical Center, Dallas, TX 75390, USA
[15]Department of Surgery, University of Michigan, Ann Arbor, MI 48109, USA
[16]Department of Cell and Developmental Biology, University of Michigan, Ann Arbor, Michigan 48109, USA.
* Correspondence: clyssiot@med.umich.edu

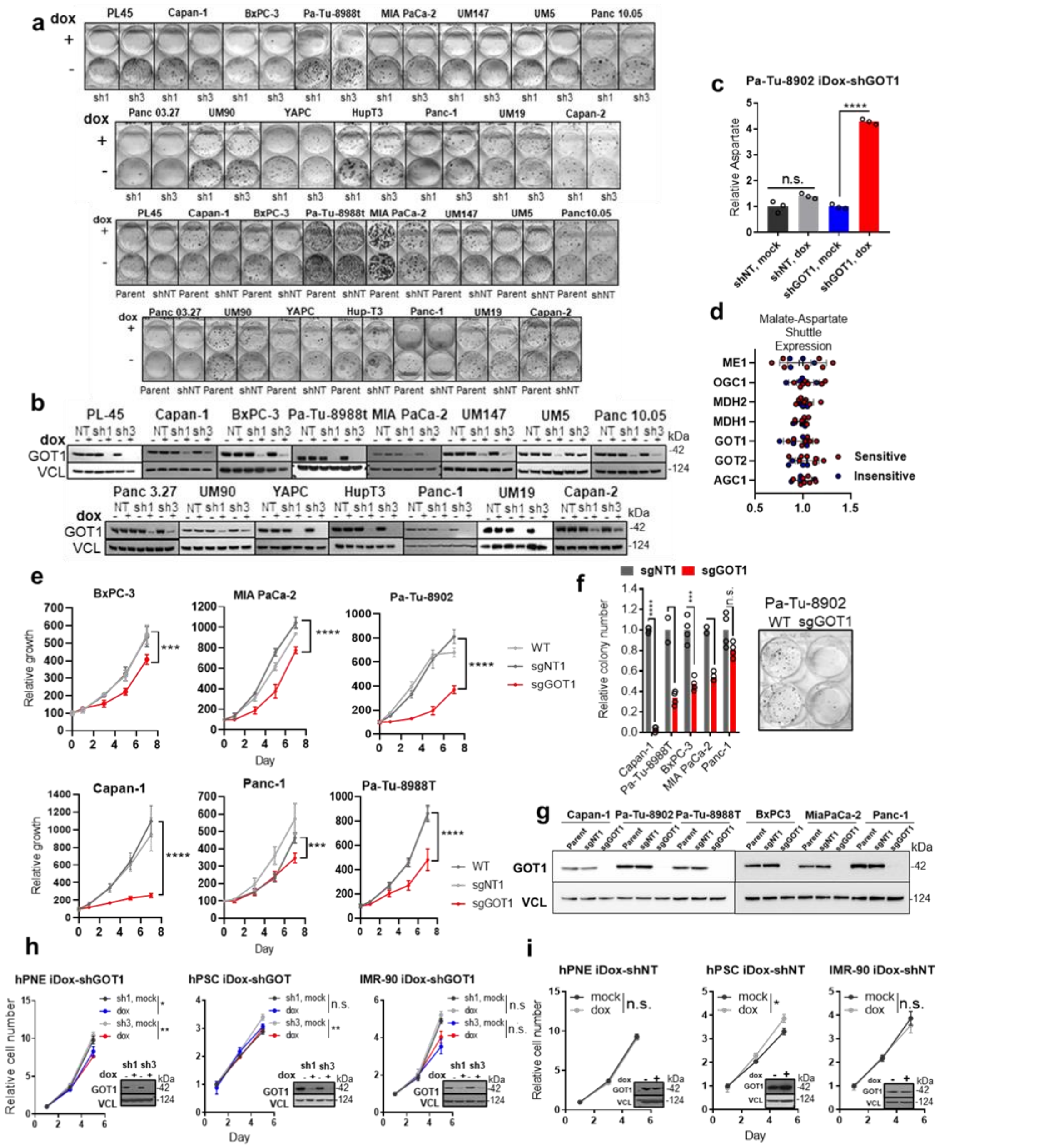


**Supplementary Figure 1.** GOT1 is dispensable in non-transformed cell lines.
**a-b)** Colony formation assays (a) and GOT1 immunoblots (b) from 1d. Blots in (b) are representative of three independent experiments. **c)** LC-MS/MS measurements of aspartate following five days of knockdown, n=3 biologically independent experiments, ****P<0.0001 idox-shGOT1. **d)** mRNA expression of malate-aspartate shuttle components in PDA cell lines from the Cancer Cell Line Encyclopedia. **e)** Proliferation (n=4 biologically independent samples); BxPC-3 ***P=0.0007; MIA PaCA-2 ****P=<0.0001; ****Pa-Tu-8902 P=<0.0001, ****Capan-1 P=<0.0001, Panc-1 ***P= 0.0001, Pa-Tu-8988T ****P= <0.0001; **f)** Colony formation Capan-1, n=4, ****P=<0.0001; Pa-Tu-8902, n=2, BxPC-3, n=4, ***P=0.0003, MIA PaCa-2, n=2; Panc-1, n=4, n.s. P=0.0950. Derived from biologically independent samples; **g)** Western blot following sgGOT1 corresponding to figures 1e-f and **Supplementary Data 1e**. Blots are representative of two independent experiments. **h-i)** Proliferation of immortalized non-transformed human cell lines normalized to day 1 for iDox-shGOT1 (h) hPNE, n=3, *P=0.0358 sh1 and **P=0.0054 sh3; hPSC, n=3, n.s. P= 0.0614 sh1 and **P=0.0072 sh3; IMR-90, n=4, n.s. P=0.0379 sh1, and n.s. P=0.0974 sh3 and iDox-shNT (i) hPNE, n=3, n.s. P=0.2783; hPSC, n=3, n.s. P=0.0126; IMR-90, n=4, n.s. P=0.2293 derived from biologically independent samples. Error bars represent mean ± SD. Two-tailed unpaired T-test or 1-way ANOVA: Non-significant P > 0.05 (n.s. or # as noted). Source data are provided as a Source Data file.

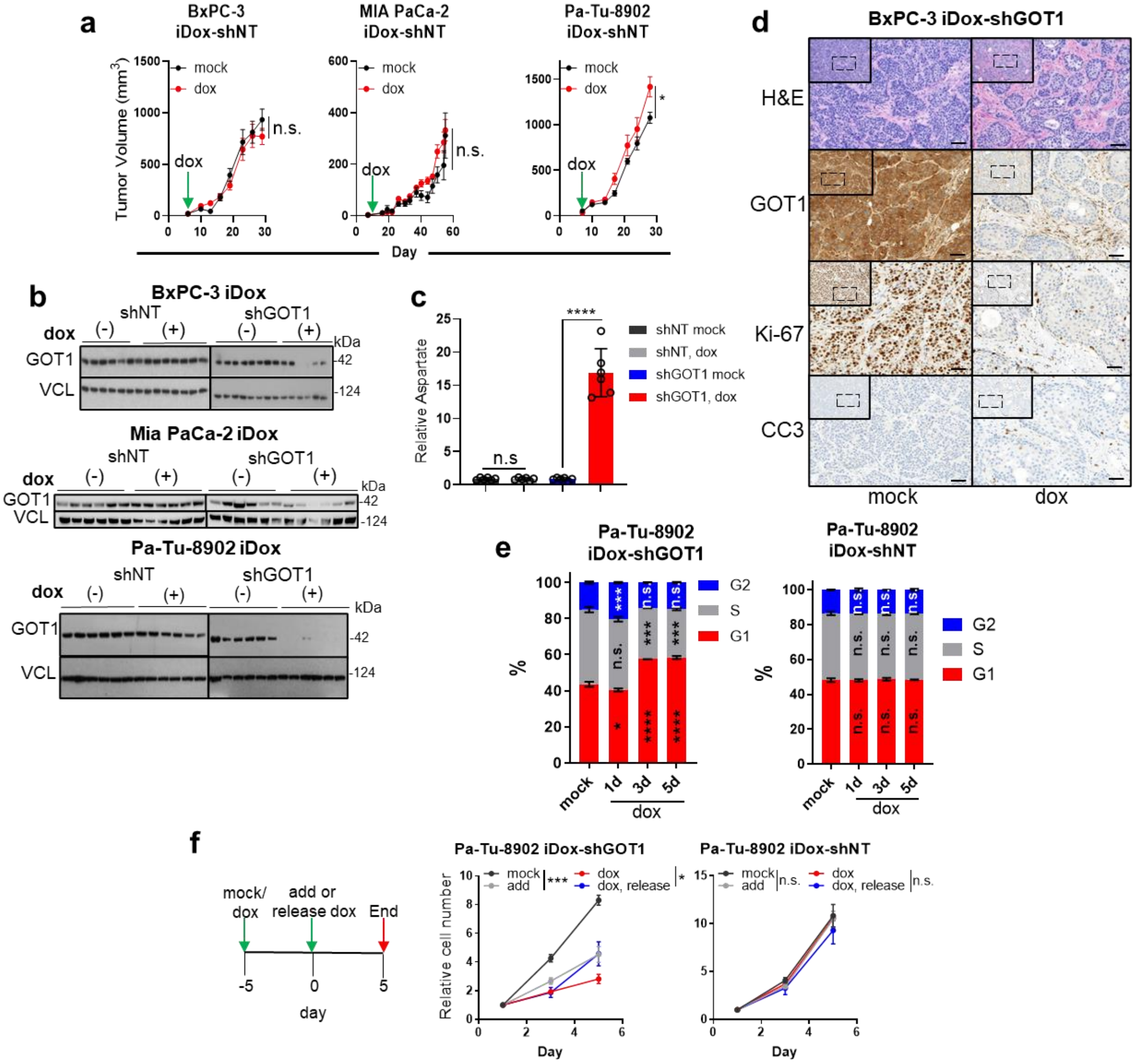


**Supplementary Figure 2.** GOT1 inhibition is cytostatic.
**a)** Growth of subcutaneous xenograft tumors containing non-targeting (NT) vectors treated with dox (red) or vehicle (black) (n= 6 tumors) corresponding to Figure 1e, BxPC3 n.s. P= 0.2254; MIA PaCA-2 n.s. P= 0.8362; Pa-Tu-8902 *P= 0.0227. **b)** Immunoblots for GOT1 from tumors in **1g** and S**upplementary Fig. 2a.** Blots are representative of two independent experiments for BxPC-3 and Pa-Tu-8902 and one independent experiment for MIA PaCa-2. **c)** LC-MS/MS measurements of aspartate taken from homogenized Pa-Tu-8902 iDox-shGOT1 tumors, n=6, n.s. P=0.9195 and ****P<0.0001. **d)** Histology of BxPC-3 iDox-shGOT1 subcutaneous xenograft tumors from vehicle-or dox-treated mice. H&E, Hematoxylin and Eosin, CC3, cleaved caspase 3. Scale bars represent 50µm. Micrographs are representative of 6 independent tumors. **e)** Cell cycle upon 1, 3, or 5 days of dox treatment. Significance values are in relation to iDox-shGOT1 mock (n=3 biologically independent samples), G2 1d ***P=0.0002, G1 1d *P=0.0264; S 3d ***P=0.0001; G1 3d ****P=<0.0001; S 5d ***P=0.0001; G1 5d ****P<0.0001. **f)** Proliferation kinetics following GOT1 knockdown. Cells were untreated (black), dox was added to untreated cells (blue), pre-treated with dox and chronically exposed to dox (grey), or released from dox pretreated cells (red). Relative cell number at day 5 normalized to day 1 is displayed, (n=3 biologically independent samples), ***P=0.0002 mock and add; *P=0.0202 dox and dox, release. Error bars represent mean ± SD or mean ±SEM in (a and d). Two-tailed unpaired T-test or 1-way ANOVA: Non-significant P > 0.05 (n.s. or # as noted). Source data are provided as a Source Data file.

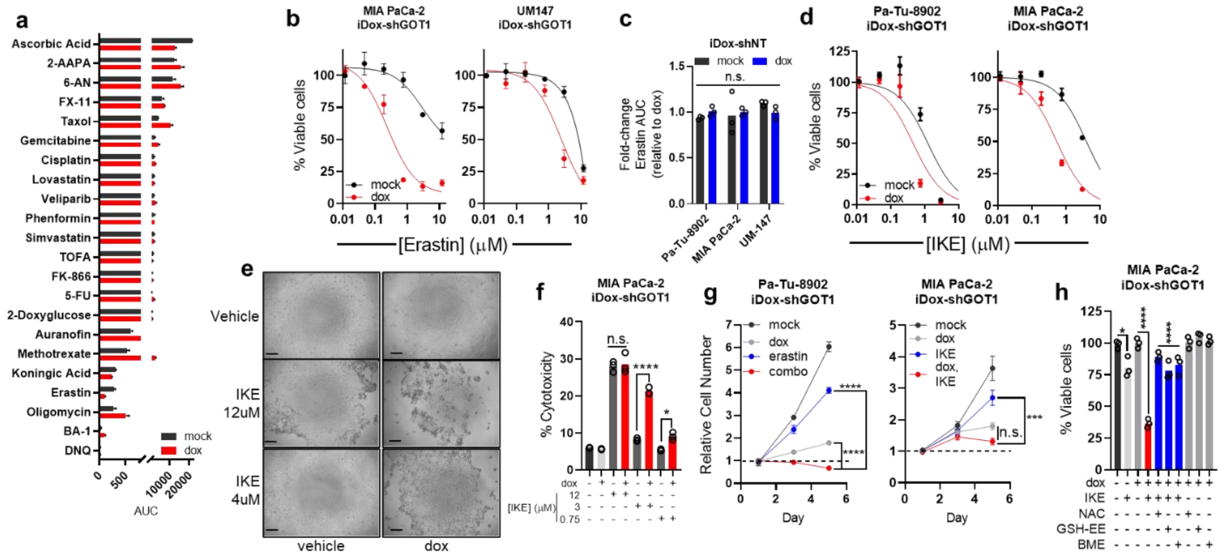

**Supplementary Figure 3.** GOT1 Inhibition sensitizes PDA to $x_c$- inhibitors.

**a)** Area under the curve (AUC) in cell viability for each compound treated for 72 hours, n=3 biologically independent samples. **b)** Cell viability dose response curves for erastin after 24 hours in iDox-shGOT1 expressing cell lines. **c)** AUC fold-change for shNT expressing cell lines, n=3 biologically independent samples. **d)** Imidazole ketone erastin (IKE) cell viability dose curves, n=3 biologically independent samples. **e)** Bright field images of Pa-Tu08982 iDox-shGOT1 cells treated with IKE for 24 hours. Representative of two independent experiments.Scale bars represent 20µm. **f)** % Cytotoxicity following GOT1 knockdown and IKE treatment for 24 hours. Cytotoxicity was measured by LDH release and normalized to a cell lysis control, n=3 biologically independent samples, ****P=<0.0001 and *P=0.0311. **g)** Proliferation after 5 days of dox treatment with the indicated conditions. 750nM of Erastin or IKE was administered on day 1 and conditions are normalized to day 1 (n=3 biologically independent samples), ****P=<0.0001 Pa-Tu-8902 and ***P=0.0005 MIA PaCa-2. **h)** Cell viability of Mia PaCa-2 iDox-shGOT1 after 5 days of dox culture then 750nM IKE co-cultured with the indicated conditions (n=3 biologically independent samples). *P=0.0104 vehicle vs IKE, ****P=<0.0001 dox vs dox/IKE, ****P=<0.0001 dox/IKE vs, dox/IKE/NAC, GSH-EE, or BME. Error bars represent mean ± SD. Two-tailed unpaired T-test or 1-way ANOVA: Non-significant P> 0.05 (n.s. or # as noted). Source data are provided as a Source Data file.

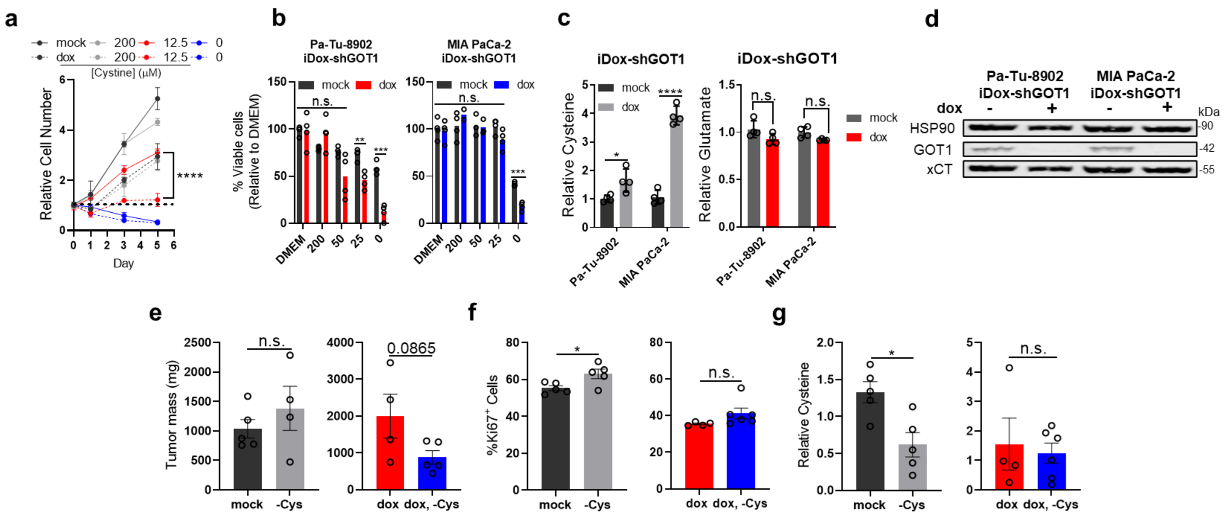


**Supplementary Figure 4**. PDA cultures require exogenous cystine for proliferation and viability.
**a)** Mia PaCa-2 iDox-shGOT1 proliferation following 5 days of GOT1 knockdown and the indicated media conditions (n=3 biologically independent experiments), ****P=<0.0001. **b)** Cell viability of Pa-Tu-8902 iDox-shGOT1 (red) and MIA PaCa-2 iDox-shGOT1 (blue) following 5 days of dox pre-treatment and 24 hours of the indicated cystine concentrations (n=4 biologically independent experiments), **P=0.002013 and ***P=0.000125 Pa-Tu-8902; ***P=0.000062 MIA PaCa-2.**c)** LC-MS/MS measures of intracellular cysteine and glutamate upon GOT1 knockdown, n=4 independent samples, Cysteine *P=0.0239 Pa-Tu-8902; ****P=<0.0001 MIA PaCa-2. **d)** xCT levels following GOT1 knockdown. Representative of 2 independent experiments. **e)** Post-treatment tumor mass (mock, n=5 tumors), (-Cys, 4), (dox, n=4), and (dox, -Cys, n=5). n.s. P=0.3873 mock vs. -Cys; n.s. P=0.0865 dox vs. dox/-Cys. **f)** Ki-67 staining in post-treatment tumors, n=5, * P=0.0294 mock vs.-Cys; n.s. P=0.1607 dox vs. dox/-Cys. **g)** Cysteine levels in tumors at endpoint, n=5, n.s. P=0.0113 mock vs.-Cys and n.s. P=0.7171 dox vs. dox/-Cys. Error bars represent mean ± SD (Figures a-c) or mean ± S.E.M (Figures e-g). Two-tailed unpaired T-test or 1-way ANOVA: Non-significant P> 0.05 (n.s. or # as noted). Source data are provided as a Source Data file.

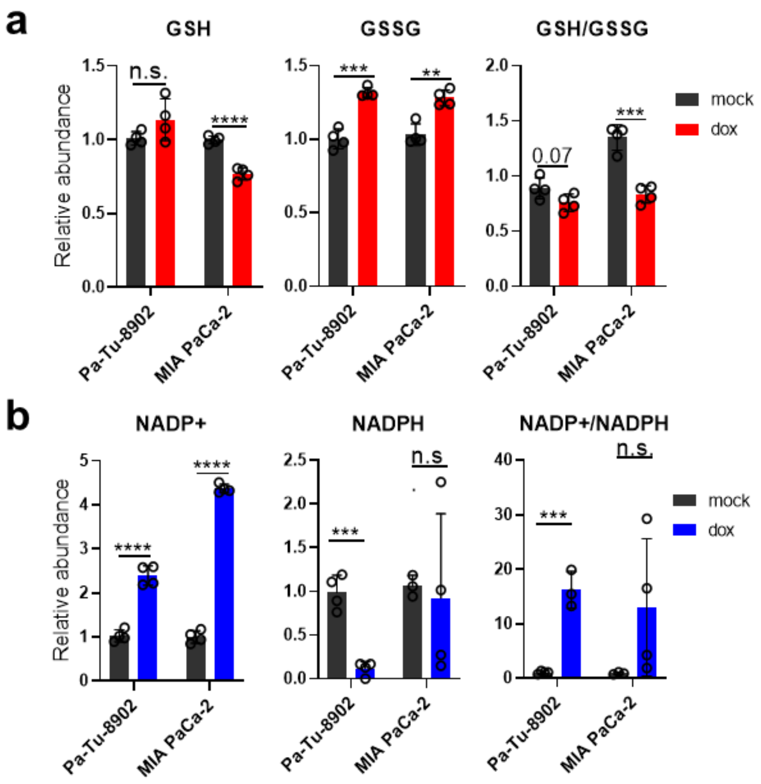


**Supplementary Figure 5.** GOT1 inhibition promotes redox stress.
**a-b)** LC-MS/MS measures of redox co-factors, n=4 biological replicates. GSH n.s. P= 0.1370 Pa-Tu8902; **** P <0.0001 MIA PaCa-2, GSSG *** P= 0.0002; ** P= 0.0015; GSH/GSSG n.s. P= 0.0774; *** P= 0.0003. NADP+ **** P= <0.0001 Pa-Tu-8902 and MIA PaCa-2; NADPH *** P= 0.0002; n.s. P= 0.8178. NADP+/NADPH *** P= 0.0003; n.s. P= 0.1663. Error bars represent mean ± SD. Two-tailed unpaired T-test: Non-significant P> 0.05 (n.s. or # as noted). Source data are provided as a Source Data file.

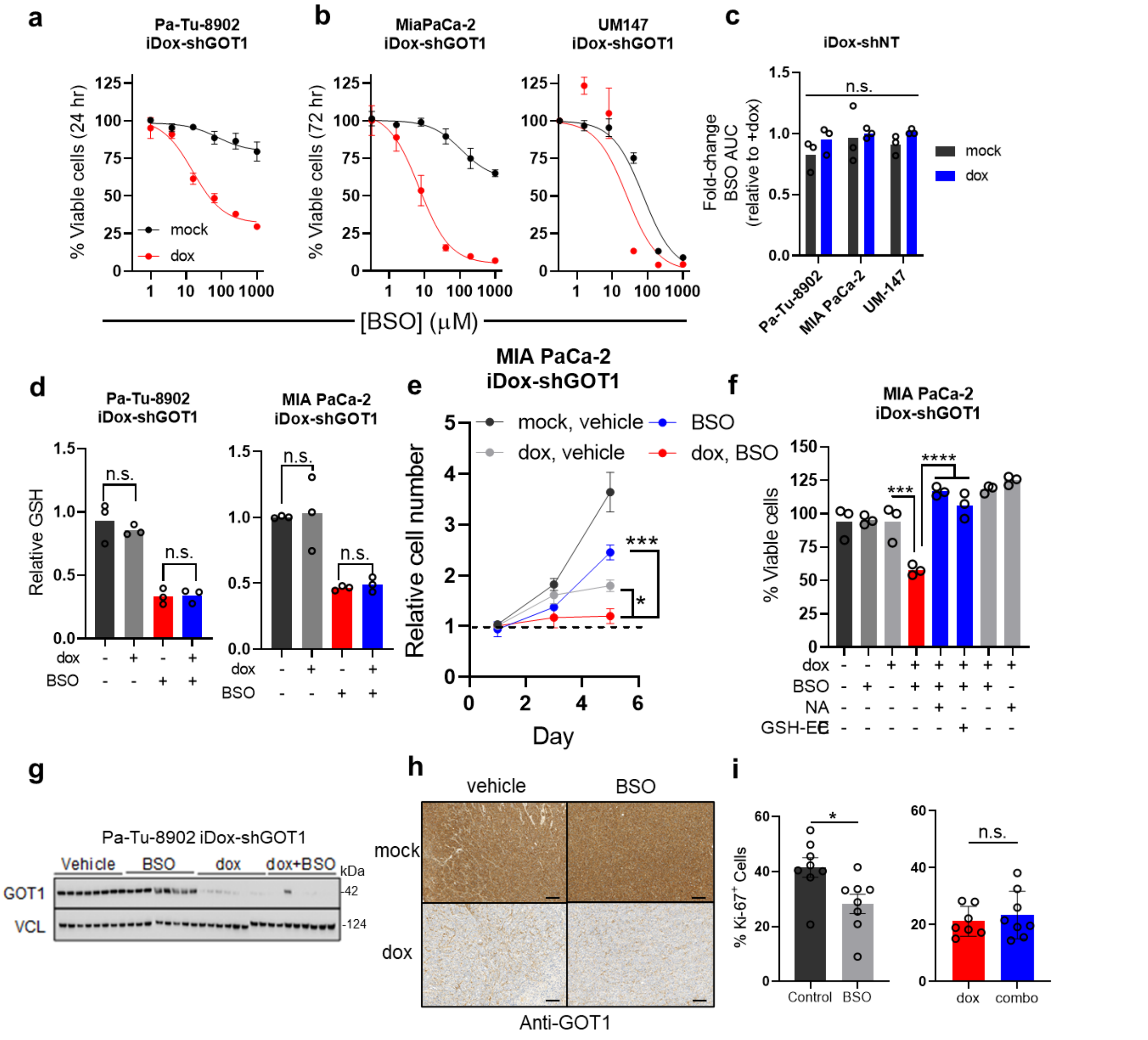


**Supplementary Figure 6.** PDA require GSH synthesis under GOT1 deficient conditions.
**a-b)** Cell viability dose response at 24 (a) and 72 hours (b), n=3 biologically independent samples representative of two independent experiments. **c)** AUC fold change in shNT matched cell lines, n=3 biologically independent samples. **d)** GSH-glo measures, n=3 biologically independent samples, n.s. P=0.7408 and P=0.9996 in Pa-Tu-8902; n.s. P=0.9947 and P= 0.9956 in MIA PaCa-2. **e)** Proliferation after 5 days of GOT1 knockdown. BSO 40μM treatment was initiated on day 1 and curves are normalized to day 1, n=3 biologically independent samples,*** P=0.0007 and * P=0.0488. **f)** Relative viability after 5 days of GOT1 knockdown and treatment with 40μM BSO or co-treatment with 0.5mM N-acetyl cysteine (NAC) 0.5mM GSH-ethyl ester (GSH-EE), n=3 biologically independent samples, *** P= 0.0006; **** P=<0.0001. **g)** Immunoblot analysis of GOT1 from tumors in Figure 3f (n=8 tumors). Representative of a single independent experiment. **h)** Immunohistochemical staining of GOT1, n=8 tumors. Scale bars represent 50μm. **i)** Quantification of Ki-67 staining in tumors from 3f, n=8 tumors, * P=0.0194. Error bars represent mean ± SD or mean ± SEM in (i). Two-tailed unpaired T-test or 1-way ANOVA: Non-significant P> 0.05 (n.s. or # as noted). Data are provided as a Source Data file.

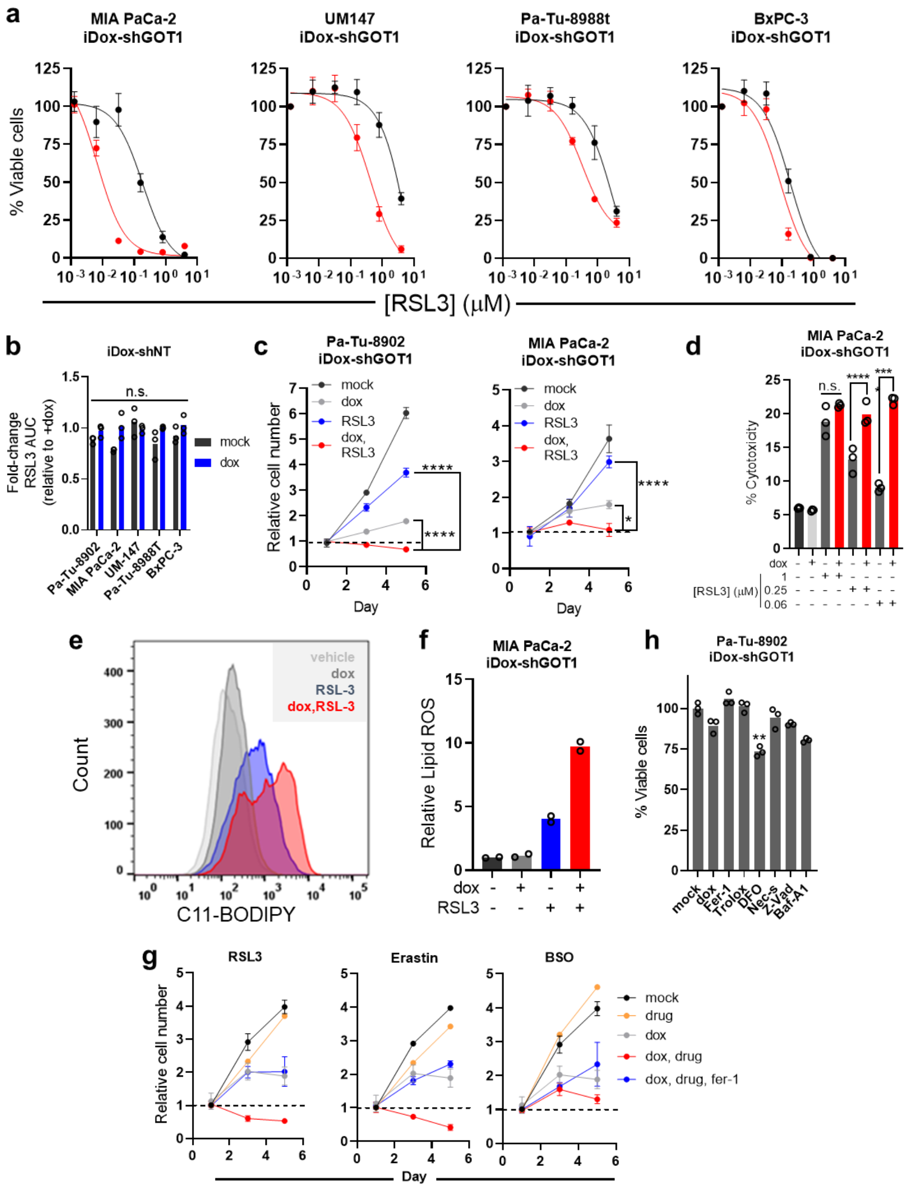


**Supplementary Figure 7.** Additional data demonstrating GOT1 inhibition sensitizes PDA to ferroptosis.
**a)** Cell viability dose response curves at 24 hours, n=3 biologically independent samples. **b)** Fold change in cell viability AUC in shNT cells, n=3 biologically independent samples. **c)** Proliferation following 5 days of knockdown and treatment with the indicated conditions. 32nM of RSL3 was administered on day 1. Cell numbers are normalized to day 1 for each condition, n=3 biologically independent samples. ****P=<0.0001. **d**) % Cytotoxicity following GOT1 knockdown and RSL3 treatment at 24 hours. Cytotoxicity was measured by LDH release and normalized to a cell lysis control, n=3 biologically independent samples. ****P=<0.0001. **e)** Distribution of Pa-Tu-8902 iDox-shGOT1 cells positive for C11-BODIPY corresponding to (4e). **f)** Fold change in viable MIA PaCa-2 iDox-shGOT1 cells positive for C-11 BODIPY, following 5 days of GOT1 knockdown. Cells were treated with the indicated conditions for 6 hours prior to measurements: vehicle (0.1% DMSO) -/+ dox (black and grey), 1µM RSL3. Data are normalized to the –dox and vehicle-treated condition, n=2 biologically independent samples. **g)** Proliferation of Pa-Tu-8902 iDox-shGOT1 following 5 days of knockdown and treatment with the indicated conditions (n=3 biologically independent samples). 32nM RSL3, 750nM Erastin, or 40µM BSO -/+ 1µM Ferrostatin-1 (Fer-1) were used. Cell numbers are normalized to day 1 for each condition, n=3 biologically independent samples. **h)** Single agent viability controls for 4f-g, n=3 biologically independent samples. DFO vs. dox **P=0.0016. Error bars represent mean ± SD. Two-tailed unpaired T-test or 1-way ANOVA. Non-significant P> 0.05 (n.s. or # as noted). Source data are provided as a Source Data file.

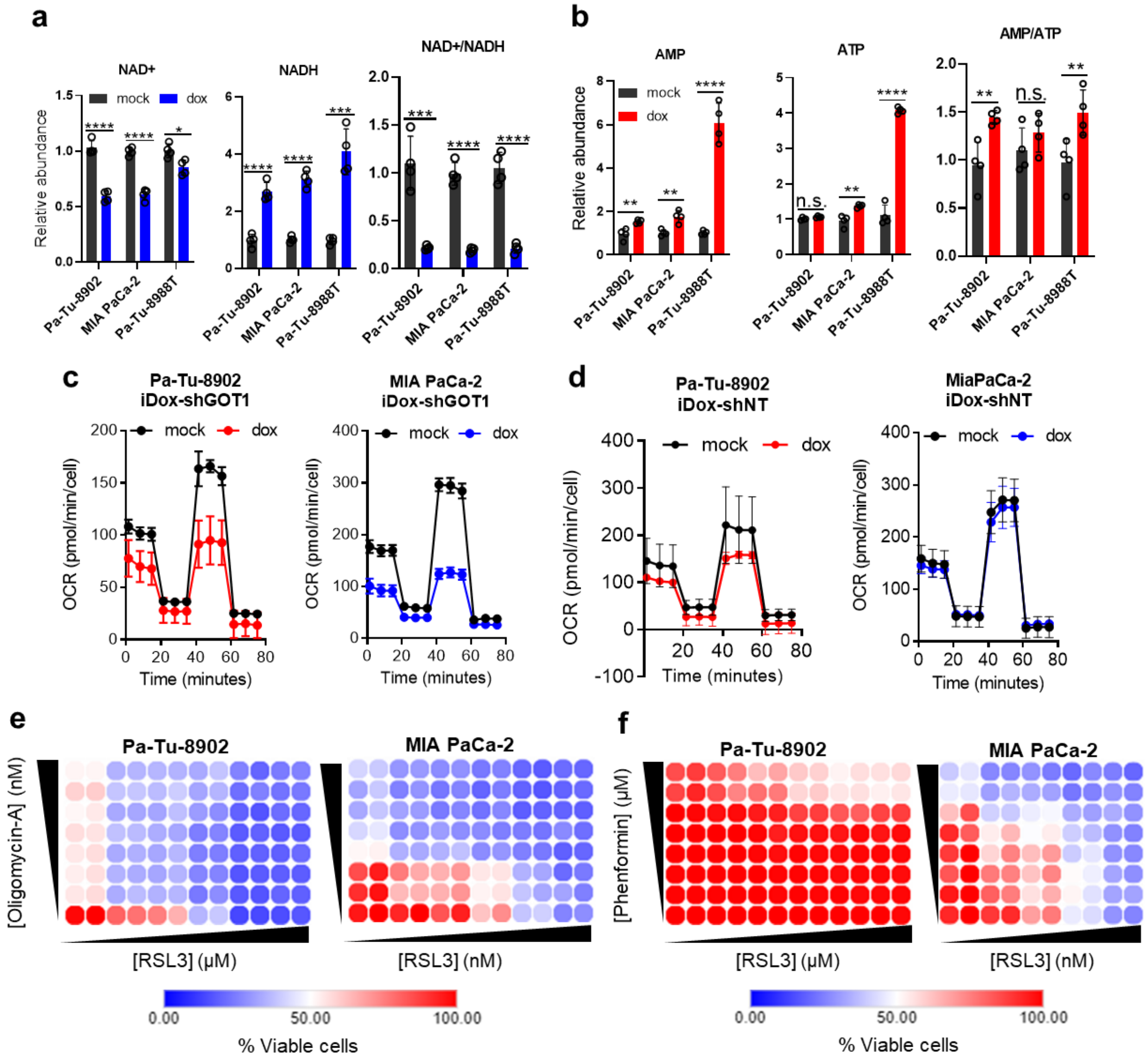

**Supplementary Figure 8.** Inhibition of mitochondrial metabolism potentiates ferroptosis

**a-b)** LC-MS/MS measurements of $NAD^+$, NADH, AMP, and ATP following 5 days of GOT1 knockdown, n=4 biologically independent experiments. NAD+ ****P= <0.0001; *P=0.0158. NADH ****P=<0.0001; ***P=0.0003. NAD+/NADH ***P= 0.0008; ****P=<0.0001. AMP **P=0.0054; **P=0.0032; ****P=<0.0001. ATP **P=0.0025; ****P=<0.0001. **c-d)** Oxygen consumption rate following GOT1 knockdown, n=3 biologically independent experiments. **e-f)** Dose response matrix of cell viability following 72 hours co-drug treatment, n=2 biologically independent experiments. Error bars represent mean ± SD. Two-tailed unpaired T-test or 1-way ANOVA: Non-significant P> 0.05 (n.s. or # as noted). Source data are provided as a Source Data file.

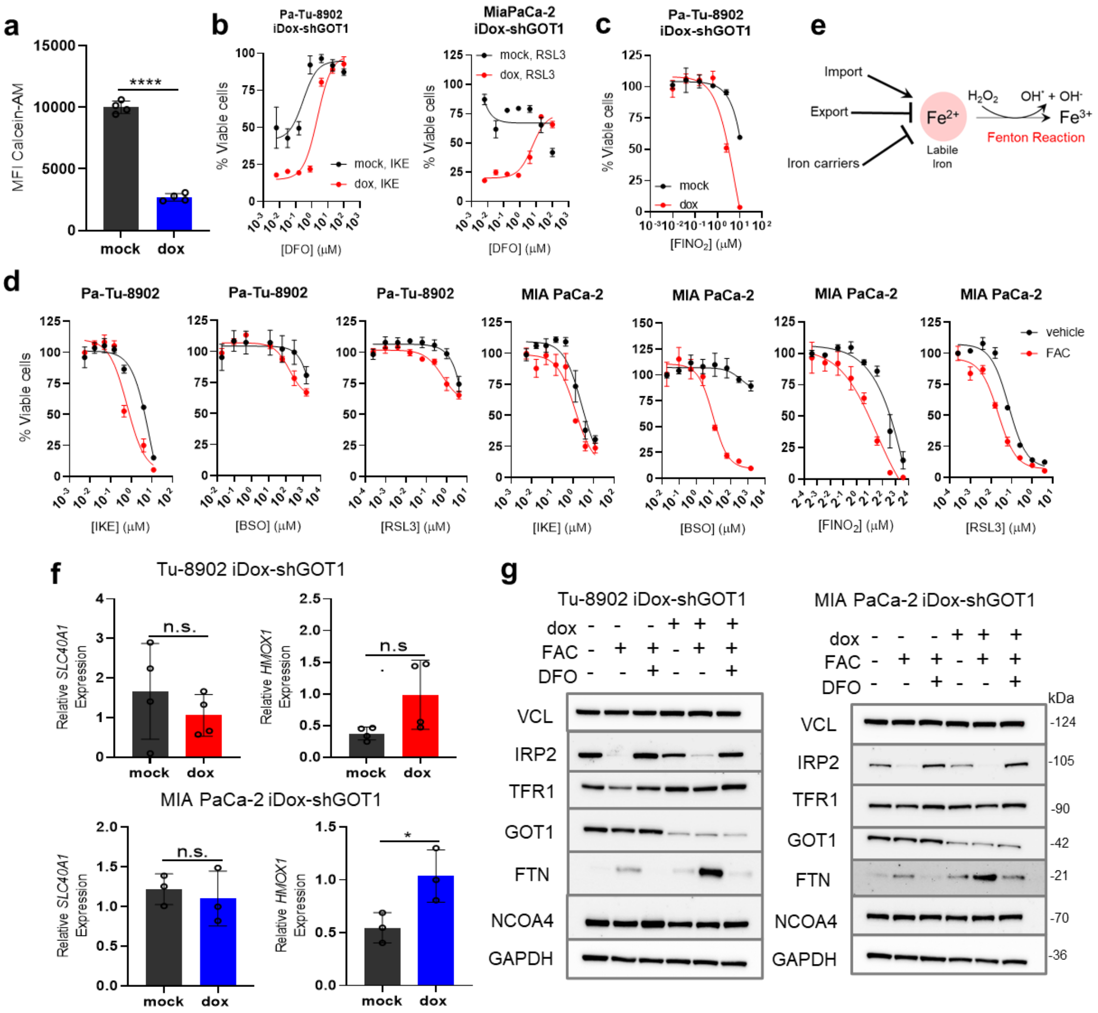


**Supplementary Figure 9.** Labile iron potentiates ferroptosis.
**a)** Calcein-AM MFI in Mia PaCa-2 iDox-shGOT1 following GOT1 knockdown, n=3 biologically independent experiments. **** P= <0.0001. **b-d)** Cell viability dose response curves of various ferroptosis inducers treated with DFO (n=3 biologically independent experiments) (b), $FINO_2$ following GOT1 knockdown (c), or 200µM of ferric ammonium citrate (FAC) (d), n=3 biologically independent experiments. **e)** Scheme of iron release mechanisms. **f)** Expression of *SLC40A1* and *HMOX1* in Pa-Tu08902 (red), n=4 biological replicates, or Mia PaCa-2 iDox-shGOT1 cells (blue), n=3 biological replicates. * P= 0.0417. **g)** Westerns of iron responsive genes following GOT1 knockdown. Representative of two independent experiments. Error bars represent mean ± SD. Two-tailed unpaired T-test or 1-way ANOVA: Non-significant P> 0.05 (n.s. or # as noted). Source data are provided as a Source Data file.

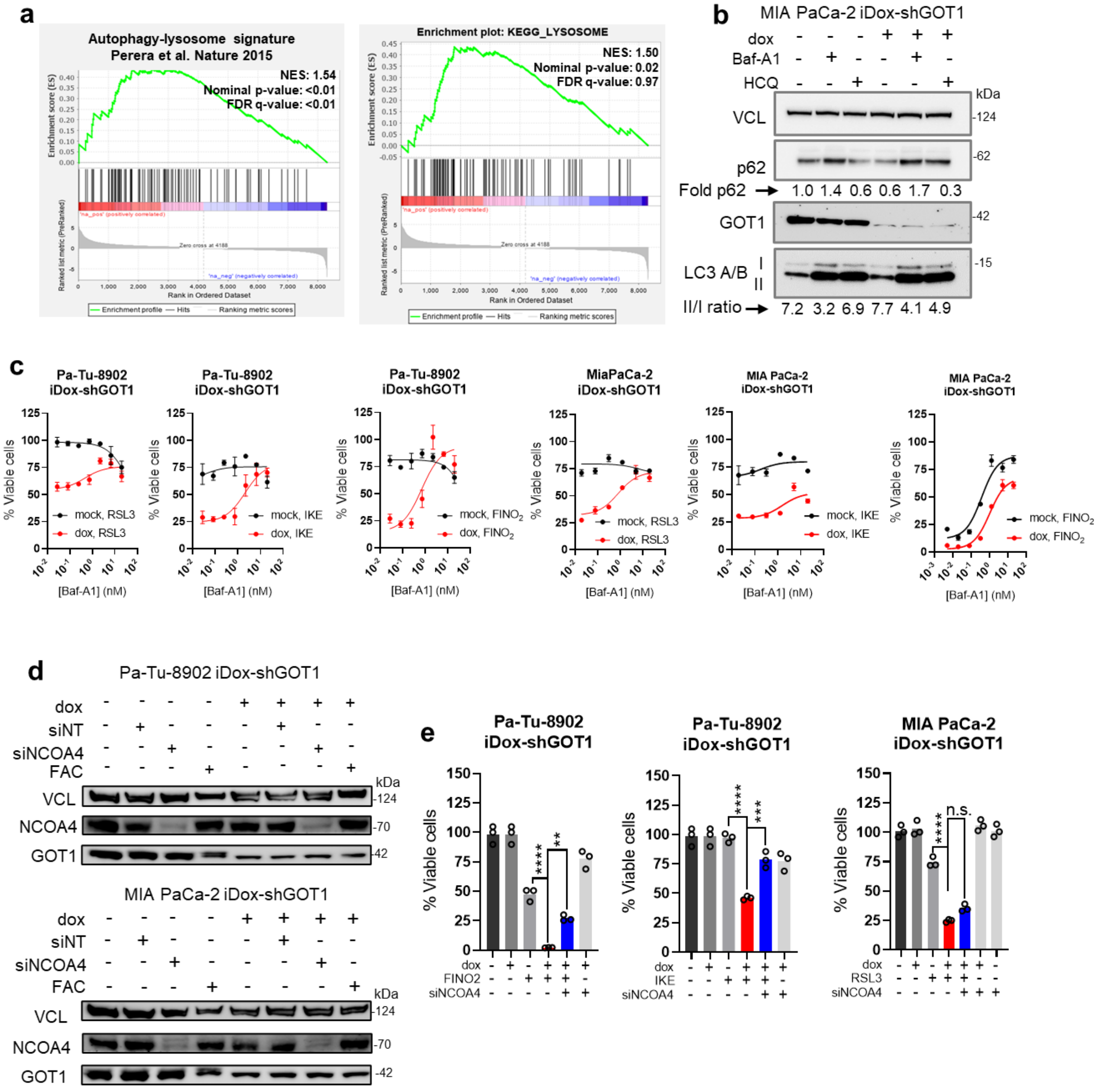


**Supplementary Figure 10.** Blocking lysosomal acidification or ferritinophagy blocks the GOT1 potentiation of ferroptosis.
**a)** Enrichment of lysosomal transcripts following GOT1 silencing in Pa-Tu-8902 iDox-shGOT1, n=3 biologically independent experiments. **b)** Western blot of autophagy markers in MIA PaCa-2. Representative of two independent experiments. **c)** Cell viability of cells co-treated with ferroptosis triggering compounds and increasing doses of Baf-A1, n=2 biologically independent experiments. **d)** Western blots analysis of NCOA4 after siNCOA4. Representative of three independent experiments. **e)** Knockdown of NCOA4 rescues GOT1-mediated ferroptosis in Pa-Tu-8902 and MIA PaCa-2, n=3 biologically independent experiments. Pa-Tu-8902 FINO2 vs. dox/FINO2 ****P<0.0001; dox/FINO2 vs. dox/FINO2/siNCOA4 ***P=0.0026. IKE vs. dox/IKE ****P=<0.0001; IKE vs. dox/IKE/siNCOA4 ***P=0.0005. MIA PaCa-2 RSL3 vs. dox/RSL3 ****P=<0.0001. Error bars represent mean ± SD. Two-tailed unpaired T-test or 1-way ANOVA: Non-significant P> 0.05 (n.s. or # as noted). Source data are provided as a Source Data file.

**Supplementary Table 1:** Primer Sequences used in this study.

| |
|---|
| hRPS21_F TCGCATCATCGGTGCCAAG |
| hRPS21_R GCCATTAAACCTGCCTGTGAC |
| hSLC40A1_F CCAAAGGGATTGGATTGTTG |
| hSLC40A1_R CCAAAGGGATTGGATTGTTCGTATTGTGGCATTCA |
| hHMOX1_F GGCAGAGGGTGATAGAAGAGG |
| hHMOX1_R AGCTCCTGCAACTCCTCAAA |

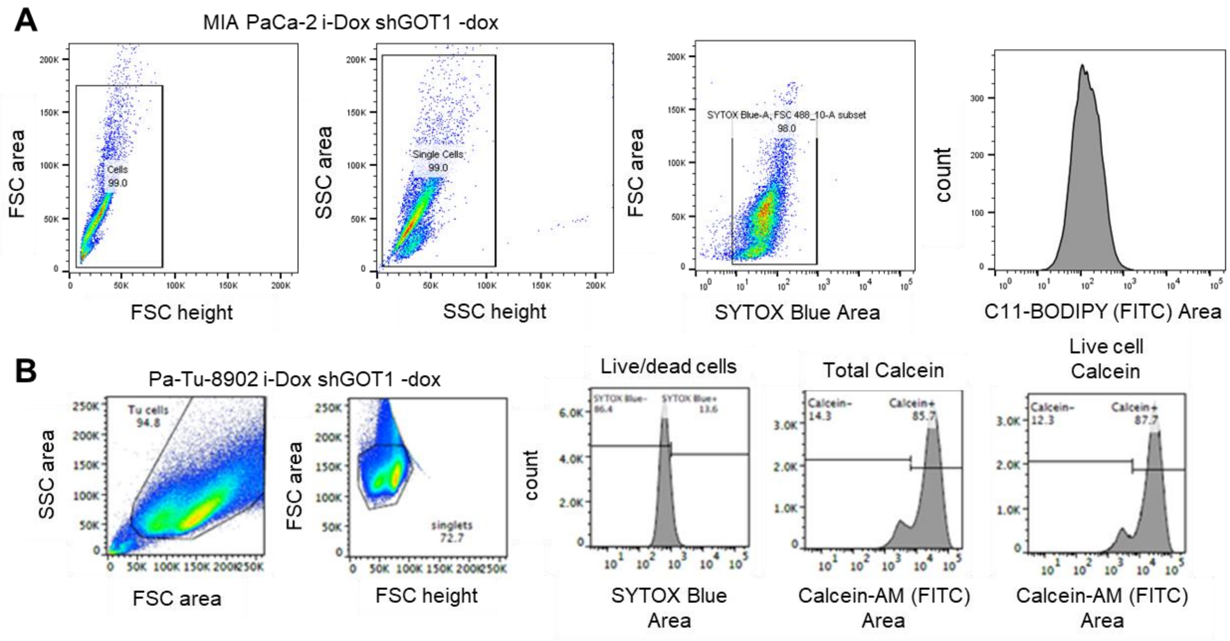


**Supplementary Figure 11.** Flow cytometry gating
**a-b)** Representative C11 BODIPY (a) and calcein-AM gating strategy (b).
