# Supplementary material for: GOT1 inhibition promotes pancreatic cancer cell death by ferroptosis
Source: Nat Commun. 2021 Aug 11;12:4860. doi: 10.1038/s41467-021-24859-2 (PMC8357841; doi:10.1038/s41467-021-24859-2)
Supplement: Supplementary file 4 — Supplemental Movies 1-10 [file 41467_2021_24859_MOESM4_ESM.zip › Supplemental Movies/Kremer, et al. (Supplmenetal Movies)_DK.pptx]

## Slide 1
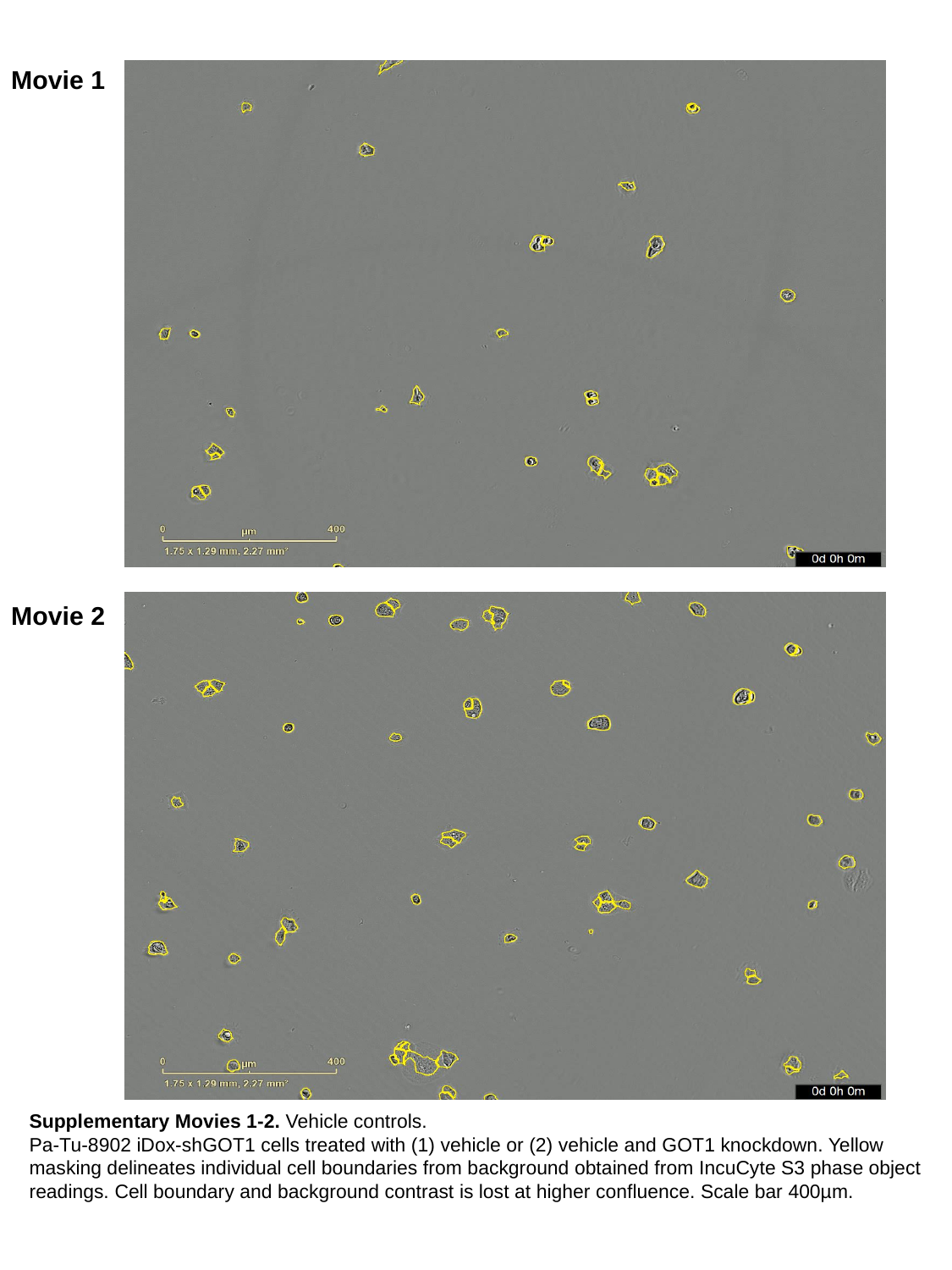

Movie 1
Movie 2
Supplementary Movies 1-2. Vehicle controls.
Pa-Tu-8902 iDox-shGOT1 cells treated with (1) vehicle or (2) vehicle and GOT1 knockdown. Yellow masking delineates individual cell boundaries from background obtained from IncuCyte S3 phase object readings. Cell boundary and background contrast is lost at higher confluence. Scale bar 400µm.

## Slide 2
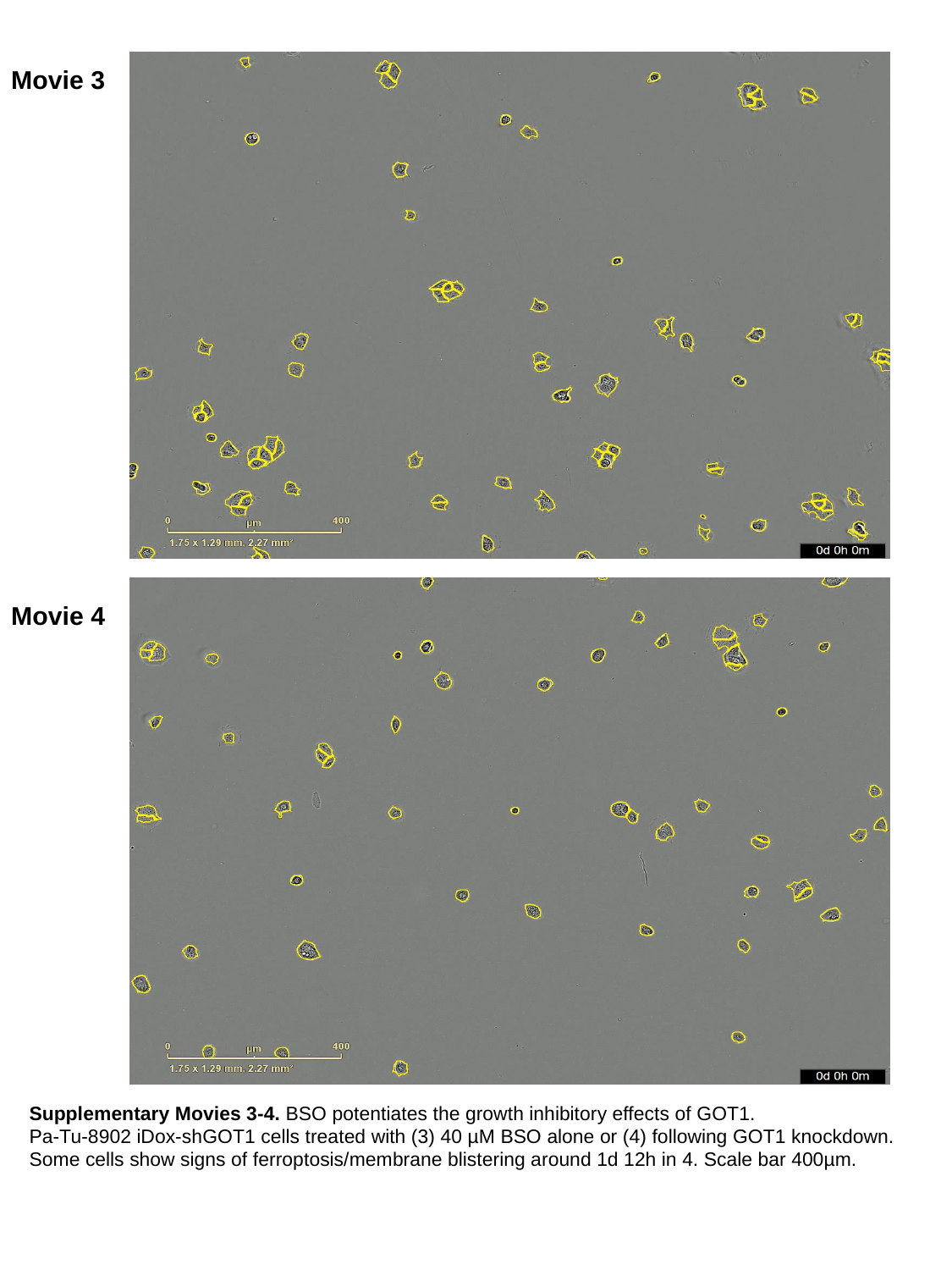

Movie 3
Movie 4
Supplementary Movies 3-4. BSO potentiates the growth inhibitory effects of GOT1.
Pa-Tu-8902 iDox-shGOT1 cells treated with (3) 40 µM BSO alone or (4) following GOT1 knockdown. Some cells show signs of ferroptosis/membrane blistering around 1d 12h in 4. Scale bar 400µm.

## Slide 3
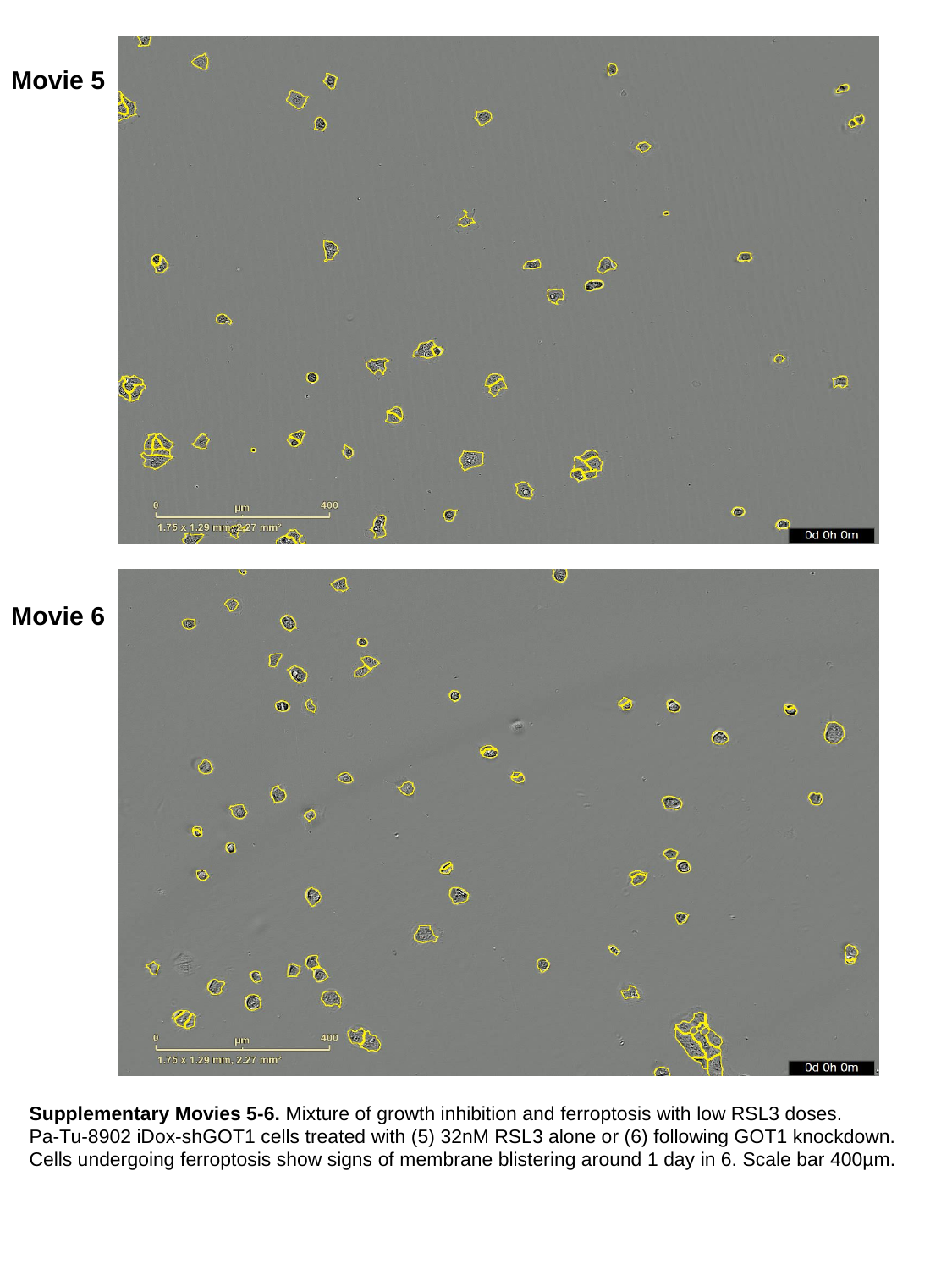

Movie 5
Movie 6
Supplementary Movies 5-6. Mixture of growth inhibition and ferroptosis with low RSL3 doses.
Pa-Tu-8902 iDox-shGOT1 cells treated with (5) 32nM RSL3 alone or (6) following GOT1 knockdown. Cells undergoing ferroptosis show signs of membrane blistering around 1 day in 6. Scale bar 400µm.

## Slide 4
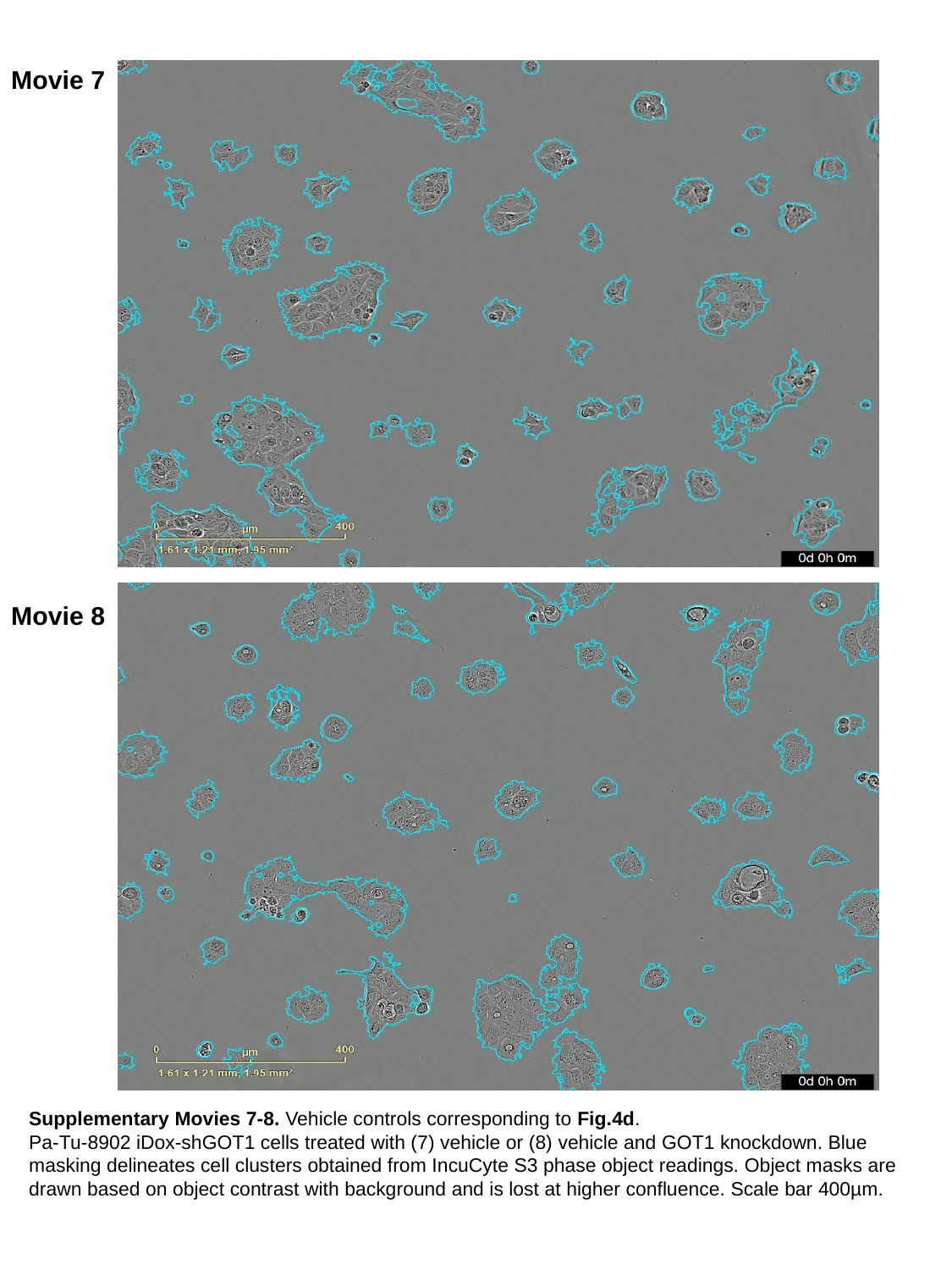

Movie 7
Movie 8
Supplementary Movies 7-8. Vehicle controls corresponding to Fig.4d.
Pa-Tu-8902 iDox-shGOT1 cells treated with (7) vehicle or (8) vehicle and GOT1 knockdown. Blue masking delineates cell clusters obtained from IncuCyte S3 phase object readings. Object masks are drawn based on object contrast with background and is lost at higher confluence. Scale bar 400µm.

## Slide 5
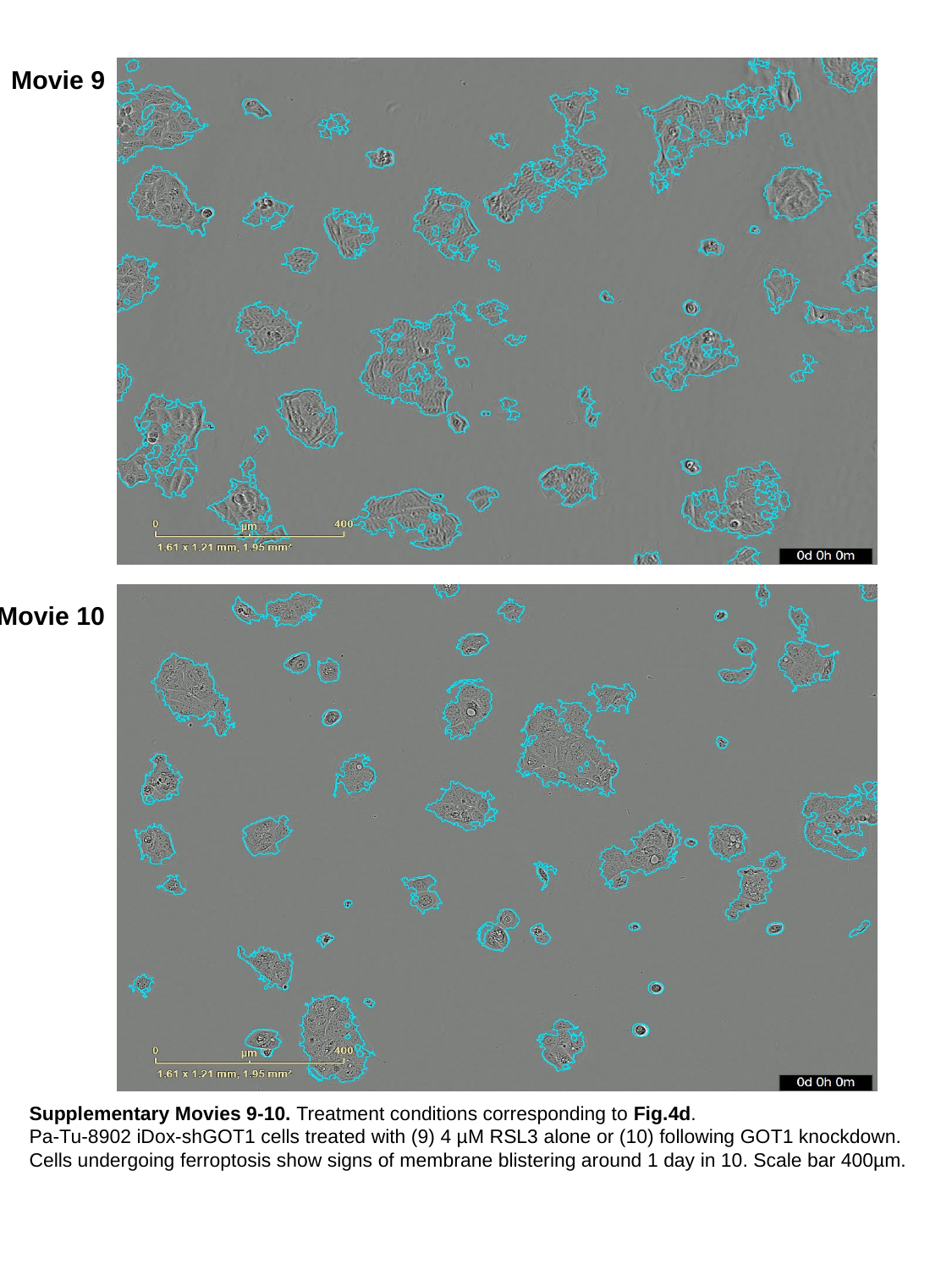

Movie 9
Movie 10
Supplementary Movies 9-10. Treatment conditions corresponding to Fig.4d.
Pa-Tu-8902 iDox-shGOT1 cells treated with (9) 4 µM RSL3 alone or (10) following GOT1 knockdown. Cells undergoing ferroptosis show signs of membrane blistering around 1 day in 10. Scale bar 400µm.
