# Supplementary material for: GOT1 inhibition promotes pancreatic cancer cell death by ferroptosis
Source: Nat Commun. 2021 Aug 11;12:4860. doi: 10.1038/s41467-021-24859-2 (PMC8357841; doi:10.1038/s41467-021-24859-2)
Supplement: Supplementary file 2 — Peer Review File [file 41467_2021_24859_MOESM2_ESM.pdf]

Editorial Note: This manuscript has been previously reviewed at another journal that is not operating a transparent peer review scheme. This document only contains reviewer comments and rebuttal letters for versions considered at Nature Communications.

REVIEWERS' COMMENTS

Reviewer #1 (Remarks to the Author):

The authors largely addressed the concerns of the reviewer. I support the publication.

REVIEWERS' COMMENTS

Reviewer #1 (Remarks to the Author):

The authors largely addressed the concerns of the reviewer. I support the publication.

We thank the reviewer for the positive comments and support for publication.
